# Trophoblast glycoprotein is a marker for efficient sorting of ventral mesencephalic dopaminergic precursors derived from human pluripotent stem cells

**DOI:** 10.1038/s41531-021-00204-8

**Published:** 2021-07-19

**Authors:** Jeong-Eun Yoo, Dongjin R. Lee, Sanghyun Park, Hye-Rim Shin, Kun Gu Lee, Dae-Sung Kim, Mi-Young Jo, Jang-Hyeon Eom, Myung Soo Cho, Dong-Youn Hwang, Dong-Wook Kim

**Affiliations:** 1 Department of Physiology, Yonsei University College of Medicine, Seoul, South Korea; 2 Severance Biomedical Research Institute, Yonsei University College of Medicine, Seoul, South Korea; 3 Department of Biomedical Science, CHA University, Sungnam, Gyeonggi-do South Korea; 4 Department of Biotechnology, College of Life Science and Biotechnology, Korea University, Seoul, South Korea; 5 S. Biomedics Co., Ltd, Seoul, South Korea; 6 Brain Korea 21 PLUS Program for Medical Science, Yonsei University College of Medicine, Seoul, South Korea

**Keywords:** Regeneration and repair in the nervous system, Pluripotent stem cells

## Abstract

Successful cell therapy for Parkinson’s disease (PD) requires large numbers of homogeneous ventral mesencephalic dopaminergic (vmDA) precursors. Enrichment of vmDA precursors via cell sorting is required to ensure high safety and efficacy of the cell therapy. Here, using LMX1A-eGFP knock-in reporter human embryonic stem cells, we discovered a novel surface antigen, trophoblast glycoprotein (TPBG), which was preferentially expressed in vmDA precursors. TPBG-targeted cell sorting enriched FOXA2^+^LMX1A^+^ vmDA precursors and helped attain efficient behavioral recovery of rodent PD models with increased numbers of TH^+^, NURR1^+^, and PITX3^+^ vmDA neurons in the grafts. Additionally, fewer proliferating cells were detected in TPBG^+^ cell-derived grafts than in TPBG^−^ cell-derived grafts. Our approach is an efficient way to obtain enriched *bona fide* vmDA precursors, which could open a new avenue for effective PD treatment.

## Introduction

Parkinson’s disease (PD) is one of the most suitable neurodegenerative disorders for cell-based therapy due to the focal degeneration of ventral mesencephalic dopaminergic (vmDA) neurons. Since the 1980s, efforts have been made to restore striatal dopamine release by engrafting fetal ventral mesencephalon tissue^1–3^. Although graft-induced recovery of motor function is possible, inconsistent outcomes and reported side effects demand improvements in cell-based treatments^4–7^. To further expand cell sources and avoid the pitfalls of using fetal tissue, including limited availability and batch-to-batch inconsistencies, increasing attention has been paid to using human pluripotent stem cells (hPSCs) due to their abundance and ability to differentiate into all cell types in the body^8,9^. Despite intensive research on the differentiation and enrichment of vmDA neurons over the past two decades^10–21^, vmDA neurons generated from hPSCs are still prone to heterogeneity, which is a significant impediment to clinical translation. Thus, obtaining more homogeneous populations of hPSC-derived vmDA cells is needed for standardization of cell sources and successful translational research.

Previous studies reported several cell surface markers (i.e., ALCAM, CD47, and CORIN) that could be used to enrich vmDA precursors^13,16,19^. However, most of these are also expressed in brain areas outside the ventral midbrain (VM). For example, activated leukocyte cell adhesion molecule (ALCAM) is a central nervous system microvascular endothelium marker that is expressed in the broad floor-plate area containing the VM and more caudal regions during early embryonic development^22^. As a result, samples obtained using anti-ALCAM antibodies contain many cell types besides vmDA precursors. It is therefore important to identify specific cell surface markers that can be used to sort vmDA precursors.

In this study, we discovered a novel surface marker, trophoblast glycoprotein (TPBG), which could be used to enrich LMX1A^+^ vmDA precursors for cell transplantation. We believe this study paves a way for the translation of hPSC-based cell therapy for PD.

## Results

### Generation of LMX1A-eGFP knock-in reporter human embryonic stem cells (hESCs)

For safe and efficient clinical application, it is desirable to remove unwanted cells (e.g., non-vmDA cells, uncommitted proliferating neural precursors, and other actively proliferating cells) as much as possible. Furthermore, enrichment of vmDA precursors would promote the efficacy of cell transplantation. Thus, we sought to uncover novel cell surface markers that could be used for sorting vmDA precursors.

**Fig. 1:**
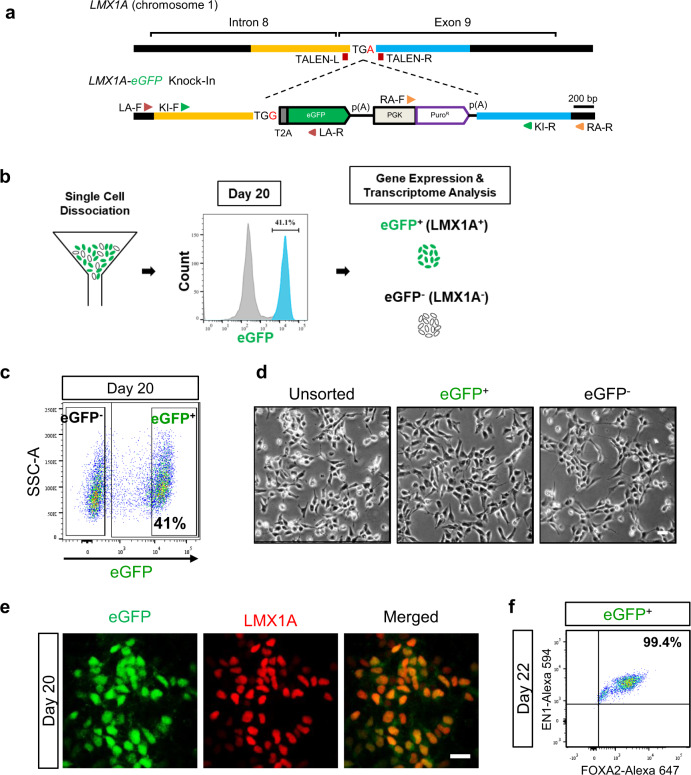
FACS isolation of eGFP^+^ cells. **a** Schematic of the generation of the LMX1A-eGFP hESC reporter line. Biallelic insertion of eGFP into exon 9 of the *LMX1A* locus was performed using a nonstop mutation (TGA > TGG) in H9 hESCs and acted as a reporter for endogenous LMX1A protein expression. **b** Schematic overview of isolation and transcriptome analysis of eGFP^+^ cells on D20. **c** The eGFP^+^ fraction was determined according to fluorescence intensity in the eGFP channel using a 488-nm laser for excitation. **d** Bright-field images of unsorted, eGFP^+^, and eGFP^−^ cells at 6 h after cells were replated. **e** eGFP^+^ cells (green) also expressed LMX1A (red). **f** Flow cytometry analysis of EN1 and FOXA2 expression in eGFP^+^ cells on D22, with three biological replicates. Scale bars, 25 μm.

To this end, we generated a reporter hESC line in which an eGFP reporter gene is inserted into the *LMX1A* locus via TALEN-mediated genome editing (Fig. 1a and Supplementary Fig. 1): Among the three clones obtained (i.e., LMX1A-R2, LMX1A-R6, and LMX1A-R7), the LMX1A-R2 cell line was used for most analyses.

The LMX1A-eGFP knock-in reporter hESCs were differentiated into vmDA precursors using a procedure modified from previous methods^10–13,23^ to obtain large numbers of vmDA cells for transplantation (Supplementary Fig. 2a). During differentiation into vmDA precursors, we visually detected eGFP fluorescence in LMX1A reporter cells on D13 post differentiation (Supplementary Fig. 2b) and found that eGFP expression displayed a similar pattern to that of *LMX1A* and two other typical vmDA progenitor markers, *EN1* and *FOXA2* (Supplementary Fig. 2c). These results indicate that eGFP expression recapitulated LMX1A expression during vmDA neuronal differentiation of LMX1A-eGFP knock-in reporter hESCs.

On D20 post differentiation, the vmDA precursor markers EN1, FOXA2, and LMX1A were detected (Supplementary Fig. 2d). Expression levels of EN1 and FOXA2 were about 95% and 86%, respectively (Supplementary Fig. 2d), suggesting efficient ventral mesencephalic differentiation. Most LMX1A-expressing cells appeared to coexpress EN1 and FOXA2, as 41% of cells were LMX1A^+^ and about 39% of cells were EN1^+^FOXA2^+^LMX1A^+^ triple-positive (Supplementary Fig. 2d).

Intriguingly, previous studies have reported that LMX1A^+^ cells comprised ~60–80% of the total cell population after differentiation^10,24,25^. The discrepancy in the percentage of LMX1A^+^ cells between our study and other reports (41% vs. 60–80%) might result from different LMX1A antibodies used for immunostaining. We used an antibody specific to LMX1A (Santa Cruz, sc-54273), while other groups used an antibody recognizing both LMX1A and LMX1B (Millipore, AB10533). To test this possibility, we performed immunostaining using the two antibodies in parallel and found that about 41% and 80% of total cells were stained with Santa Cruz and Millipore antibodies, respectively (Supplementary Fig. 2e). The percentage of EN1^+^FOXA2^+^LMX1A/B^+^ triple-positive cells among the total cell population was ∼75% when the Millipore antibody was used for immunostaining (Supplementary Fig. 2f).

### Isolation and characterization of eGFP (LMX1A)-expressing vmDA precursors

FACS was performed on D20 after vmDA differentiation of LMX1A-eGFP hESCs to collect eGFP^+^ and eGFP^−^ fractions separately (Fig. 1b). Approximately 41% of total cells were eGFP^+^ on D20 post differentiation (Fig. 1c). Both eGFP^+^ and eGFP^−^ cells showed similar morphologies (Fig. 1d). Immunocytochemistry and flow cytomety showed that most eGFP^+^ cells expressed LMX1A, EN1, and FOXA2 (Fig. 1e and f). To assess the progenitor state of sorted cells, neural and proliferative markers were examined two days after FACS. NESTIN^+^, SOX2^+^, and Ki67^+^ cells were observed in all three groups (i.e., unsorted, eGFP^+^, and eGFP^−^), indicating that sorted cells maintained neural and proliferative capacities (Supplementary Fig. 3). To ensure that eGFP^+^ precursors were indeed cycling, D20 precursors were subjected to cell cycle analysis. In eGFP^+^ cell cultures, 38.5 ± 3.9% of viable cells were in the G0/G1 phase, 49.5 ± 6.2% were in the S phase, and 6 ± 2.7% were in the G2/M phase (Supplementary Fig. 4a). Consistently, immunocytochemistry of unsorted cells showed that eGFP and cell cycle markers (i.e., Ki67 and PCNA) were largely coexpressed, whereas only a few G2/M-phase markers (e.g., PH3) were detected in eGFP^+^ cells (Supplementary Fig. 4b).

To further define the characteristics of eGFP^+^ precursors, vmDA lineage and regional markers were compared between eGFP^+^ and eGFP^−^ groups. Significant upregulation of vmDA-specific genes (i.e., *EN1*, *FOXA1*, *FOXA2*, *LMX1A*, and *LMX1B*) was observed in the eGFP^+^ group. Conversely, a serotonergic precursor-specific gene (i.e., *NKX2.2*) and red nucleus progenitor-specific genes (i.e., *SIM1*, *LHX1*, and *NKX6.1*) were downregulated relative to the unsorted and eGFP^−^ groups (Supplementary Fig. 5).

eGFP^+^ cells were further differentiated to generate mature vmDA neurons. When compared with unsorted and eGFP^−^ cell populations, eGFP^+^ cell population contained significantly enriched vmDA precursors that differentiated into TH^+^, NURR1^+^, and PITX3^+^ neurons and released more dopamine (Supplementary Fig. 6a and b).

These findings, altogether, indicate that the eGFP^+^ cells recapitulated the characteristics of vmDA precursors and were able to differentiate into mature vmDA neurons.

### Transcriptome analysis to identify cell surface markers specific to vmDA precursors

**Fig. 2:**
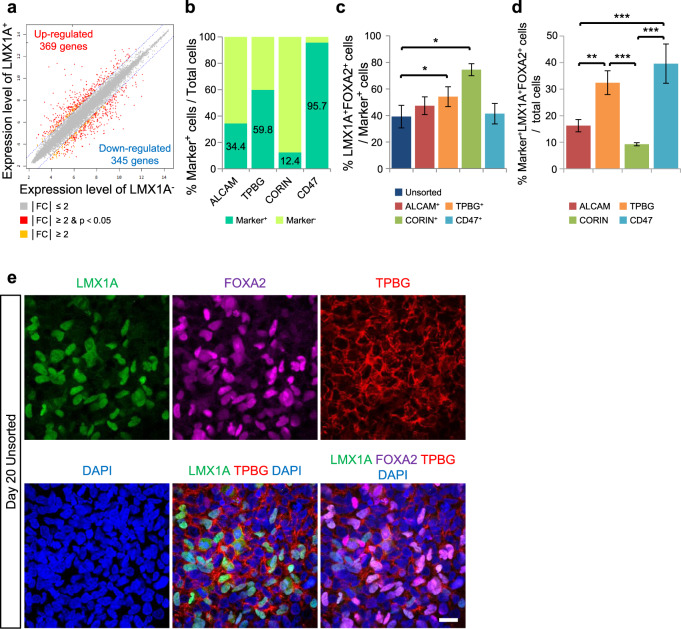
Identification of TPBG as a novel marker for sorting vmDA precursors from hESCs. **a** Comparison of gene expression profiles between eGFP^+^ (LMX1A^+^) and eGFP^−^ (LMX1A^−^) cells among vmDA precursors on D20. **b** The ratio of separated cells via MACS targeted for each marker (ALCAM, TPBG, CORIN, and CD47) on D20 with three biological replicates. **c** Flow cytometry analysis of LMX1A^+^ and FOXA2^+^ cells in unsorted, ALCAM^+^, TPBG^+^, and CD47^+^ populations on D20 with five biological replicates. *p < 0.05, one-way ANOVA with Bonferroni’s multiple-comparison tests. **d** Quantification of LMX1A^+^FOXA2^+^Marker^+^ triple-positive cells from the total population of differentiated cells on D20 with three independent experiments. **p < 0.01, ***p < 0.001, one-way ANOVA with Bonferroni’s multiple-comparison tests. **e** Representative fluorescent images for the expression of LMX1A (green), FOXA2 (magenta), and TPBG (red) in vmDA precursors on D20. DAPI (blue) was used as a counterstain. Scale bar, 25 μm.

Identifying a putative cell surface marker would improve the chances of successful stem cell therapy. To unveil vmDA precursor-specific surface markers suitable for cell sorting, we performed comparative microarray analysis of eGFP^+^ and eGFP^−^ cells. We identified 369 genes that were preferentially expressed in eGFP^+^ cells and 345 genes that were downregulated in eGFP^+^ cells (Fig. 2a). As expected, *LMX1A* was the most upregulated gene in eGFP^+^ cells relative to eGFP^−^ cells (153-fold) (Supplementary Fig. 7). On the other hand, genes related to glial and early neural precursor cells, such as *OLIG1, OLIG2, PAX6*, and *SOX1*, were more abundantly expressed in eGFP^−^ cells (Supplementary Fig. 7). This is consistent with our immunocytochemistry finding that fewer SOX1^+^ cells were detected in the eGFP^+^ cell population than in the eGFP^−^ cell population (Supplementary Fig. 3).

Among the 369 genes upregulated in eGFP^+^ cells relative to eGFP^−^ reference cells (≥2-fold change), 53 candidate genes encoding cell membrane proteins with extracellular domains were identified (Supplementary Table 1). These 53 transmembrane targets included several genes previously identified in mouse vmDA precursors (i.e., *Corin*, *Clstn2*, *Kitlg*, *Plxdc2*, *Pcdh7*, *Ferd3l*, *Frem1*, *Alcam*, and *Notch2*), thus confirming our microarray results^13,16,18,26^. Commercially available antibodies targeting only 18 out of the 53 transmembrane proteins were found for MACS purposes (Supplementary Table 1, asterisk). Of these 18 candidates, MACS targeting ALCAM, TPBG, CORIN, and CD47 resulted in 34.4%, 59.8%, 12.4%, and 95.7% of marker-positive cells out of the total number of differentiated cells, respectively (Fig. 2b). Approximately 47.3%, 54.2%, 74.5%, and 41.4% of ALCAM^+^, TPBG^+^, CORIN^+^, and CD47^+^ cell populations, respectively, consisted of LMX1A^+^FOXA2^+^ double-positive cells (Fig. 2c). Therefore, MACS targeting ALCAM, TPBG, CORIN, and CD47 allowed us to obtain approximately 16.3%, 32.4%, 9.3%, and 39.6%, respectively, of LMX1A^+^FOXA2^+^ double-positive cells from the total population of differentiated cells on D20 (Fig. 2d).

Although ALCAM, CORIN, and CD47 have been used as vmDA cell surface markers^13,16,19^, TPBG has never been described in the context of vmDA development in rodents or humans. We found that TPBG was extensively expressed in the vmDA precursor culture population (i.e., on D20 during vmDA differentiation of hPSCs) (Fig. 2e).

**Fig. 3:**
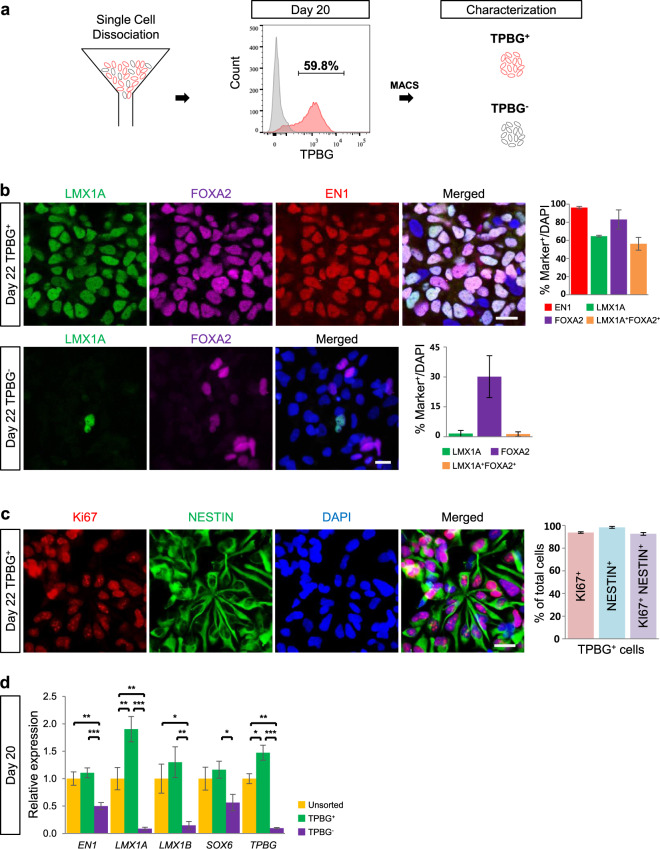
Characterization of TPBG^+^ cells. **a** Schematic overview of isolation and characterization of TPBG^+^ cells on D20. **b** Representative immunofluorescence images of TPBG^+^ cells and TPBG^−^ cells expressing LMX1A (green), FOXA2 (magenta), and EN1 (red) two days after MACS. Cells were counterstained with DAPI (blue). The percentages of EN1^+^, LMX1A^+^, FOXA2^+^, and LMX1A^+^FOXA2^+^ double-positive cells out of the total cell population are shown in the graphs. Data are the mean ± SD of three independent experiments. **c** Representative immunofluorescence images of TPBG^+^ cells targeting Ki67 (red), NESTIN (green), and DAPI (blue) (*left*). The percentages of NESTIN^+^, Ki67^+^, and NESTIN^+^Ki67^+^ double-positive cells in the total cell population are shown in this graph. Data are shown as mean ± SD of three independent experiments. **d** Comparison of relative expression of vmDA lineage markers and regional identity markers (i.e., *EN1*, *LMX1A*, *LMX1B*, and *SOX6*) on D20 among unsorted, TPBG^+^, and TPBG^−^ cell populations. Values for unsorted cells were arbitrarily set to 1. Data are shown as mean ± SEM of three biological replicates. *p < 0.05, **p < 0.01, ***p < 0.001, one-way ANOVA with Bonferroni’s multiple-comparison tests. Scale bars, 25 µm. MACS magnetic-activated cell sorting.

To verify that TPBG marks vmDA precursors, vmDA lineage markers were observed in TPBG^+^ cells: MACS-isolated TPBG^+^ cells were positive for vmDA precursor markers (i.e., EN1, FOXA2, and LMX1A) (Fig. 3a and b). However, TPBG^+^ cells still maintained neural stem cell characteristics (i.e., NESTIN^+^ and Ki67^+^) (Fig. 3a). RNA expression analysis on D20 post differentiation also showed that TPBG^+^ cells expressed significantly more ventral dopaminergic marker genes (i.e., *EN1*, *LMX1A*, *LMX1B*, and *SOX6*) than TPBG^−^ cells (Fig. 3d). By contrast, less LMX1A and FOXA2 expression were observed in TPBG^−^ cells than in TPBG^+^ cells (Fig. 3b).

**Fig. 4:**
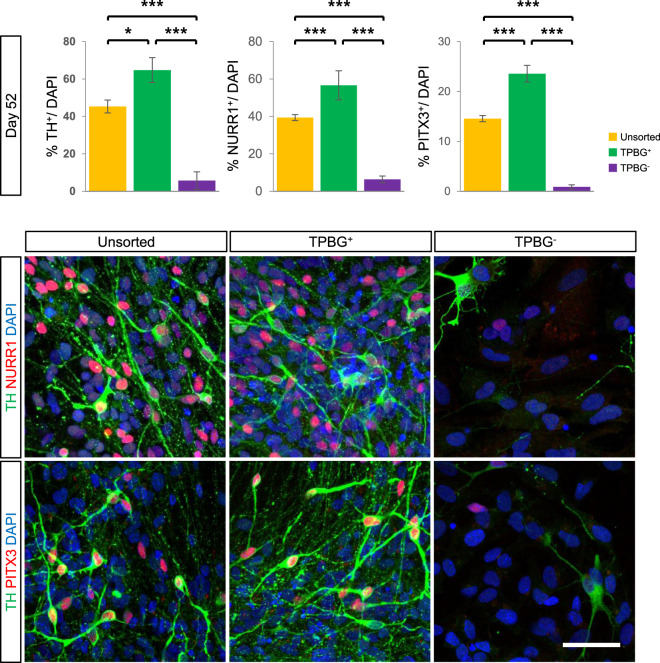
Terminal differentiation of TPBG^+^ cells. Quantification of TH^+^, NURR1^+^, and PITX3^+^ cells after terminal differentiation of unsorted, TPBG^+^, and TPBG^−^ cells on D52. Data are shown as mean ± SD of nine independent experiments. *p < 0.05, **p < 0.01, ***p < 0.001, one-way ANOVA with Bonferroni’s multiple-comparison tests (*top*). TH (green), NURR1 (red), and PITX3 (red) were analyzed by immunofluorescence staining in terminally differentiated unsorted, TPBG^+^, and TPBG^−^ cells on D52. DAPI (blue) detected total cell populations. Scale bar, 50 μm.

After maturing into vmDA neurons in vitro, significantly more neurons expressing vmDA neuronal markers, such as TH and PITX3, were detected in the TPBG^+^ group than in the TPBG^−^ and unsorted groups (Fig. 4). Together, these results indicate that TPBG could be used as a novel surface marker that specifically labels and enriches vmDA precursors.

### Transplantation of TPBG-sorted vmDA precursors into a rodent PD model

**Fig. 5:**
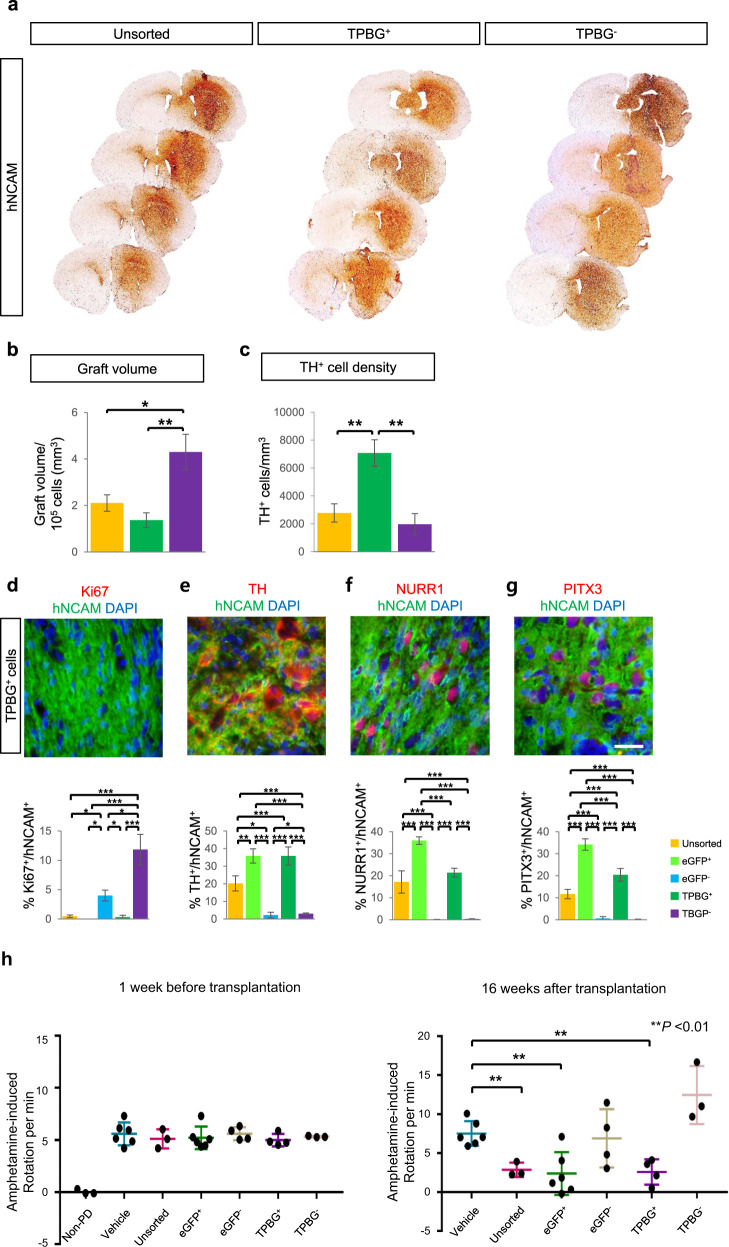
Transplantation of TPBG^+^ vmDA precursors into rodent PD models. **a** At 16 weeks after transplantation, the TPBG^+^ vmDA precursor grafts within the host striatum were visualized by hNCAM staining. **b** Graft volume of each group (unsorted, TPBG^+^, and TPBG^−^) based on immunostaining for hNCAM normalized to 100,000 grafted cells. **c** DA density in grafts from each group (TH^+^ cells/mm^3^). **d–g** Expression of hNCAM (green), Ki67 (red), TH (red), NURR1 (red), and PITX3 (red) in grafts containing TPBG^+^ cells at 16 weeks after transplantation. DAPI (blue) was used as a counterstain. Quantification of Ki67^+^, TH^+^, NURR1^+^, and PITX3^+^ cells in the grafts containing unsorted cells (*n* = 3), eGFP^+^ cells (LMX1A^+^ cells; *n* = 3), eGFP^−^ cells (LMX1A^−^ cells; *n* = 4), TPBG^+^ cells (*n* = 4), and TPBG^−^ cells (*n* = 4) at 16 weeks. *p < 0.05, **p < 0.01, ***p < 0.001, one-way ANOVA with Bonferroni’s multiple-comparison tests. **h** Amphetamine-induced rotation tests (Non-PD group, *n* = 3, vehicle, *n* = 6; unsorted, *n* = 3; eGFP^+^, *n* = 6; eGFP^−^, *n* = 4; TPBG^+^, *n* = 4; TPBG^−^, *n* = 4) one week before (*left panel*) and 16 weeks after (*right panel*) cell transplantation. **p < 0.01, unpaired Student’s *t*-tests. Data are shown as mean ± SD. Scale bar, 25 µm (**d**–**g**).

To investigate the therapeutic advantages of TPBG^+^ vmDA precursors in vivo relative to unsorted and TPBG^−^ cells, precursors from each group were transplanted into the striatum of 6-OHDA-lesioned rats. Histological analysis performed 16 weeks after transplantation showed the shape of grafts in the striatum. Total immunoreactive areas of hNCAM and graft volumes were larger in the TPBG^−^ group than those in the unsorted and TPBG^+^ groups (Fig. 5a and b). The density of TH^+^ cells in the grafts was significantly higher in TPBG^+^ grafts than those in unsorted and TPBG^−^ grafts (Fig. 5c). TH neurons extended their processes into the striatum surrounding the TPBG^+^ graft and formed connections with other neurons. DA neurons in the graft were stained with synapsin 1 on the cell membrane and on the processes in a punctate form (Supplementary Fig. 8a and b, arrowheads).

Strikingly, Ki67^+^hNCAM^+^ double-positive cells were frequently detected in TPBG^−^ grafts, but not in TPBG^+^ grafts (Fig. 5d). These data supported the need for additional sorting for safety purposes (i.e., preventing potential tumor formation). We also obtained similar results using antibodies that recognize human nuclear antigen (HNA) instead of hNCAM (Supplementary Fig. 9).

The cellular composition of the grafts was analyzed by targeting TH, NURR1, PITX3, and hNCAM (Fig. 5e–g, and Supplementary Fig. 10). LMX1A^+^ and TPBG^+^ groups were rich in TH^+^ dopaminergic neurons compared with unsorted, LMX1A^−^, and TPBG^−^ groups (Fig. 5e). The percentage of TH^+^ cells in TPBG^+^ group was approximately 35.82 ± 5.18% (Fig. 5e). When compared with unsorted, LMX1A^−^, and TPBG^−^ grafts, LMX1A^+^ and TPBG^+^ grafts had higher numbers of NURR1^+^hNCAM^+^ and PITX3^+^hNCAM^+^ dopaminergic neurons (Fig. 5f and g). In addition, cells positive for GIRK2^+^, an A9 dopaminergic neuron marker, were more abundant in TPBG^+^ grafts (29.2 ± 3.14% of total engrafted cells) than in TPBG^−^ grafts (Supplementary Fig. 11a and b). These data suggest that A9 neurons are enriched by sorting with a TPBG surface antigen.

Many human non-DA (TH^−^HNA^+^) cells in TPBG^+^ grafts were found to be neurons (TUJ1^+^) and astrocytes (GFAP^+^) (Supplementary Fig. 12a and b). Serotonergic (5-HT^+^hNCAM^+^) and glutamatergic (GLU^+^HNA^+^) neurons were hardly detected in TPBG^+^ grafts (Supplementary Fig. 13a and b).

Consistent with the histological results, rats transplanted with eGFP^−^ (LMX1A^−^) or TPBG^−^ cells did not show any functional improvement in the amphetamine-induced rotation compared with vehicle-injected 6-OHDA-lesioned rats (Fig. 5h). However, rats transplanted with unsorted, eGFP^+^ (LMX1A^+^), or TPBG^+^ cells showed significant motor function improvement at 16 weeks post transplantation (Fig. 5h).

Together, these results indicate that hESC-derived TPBG^+^ vmDA precursors share advantages of LMX1A^+^ precursors in improving motor function in vivo. It is also important to note that the TPBG^+^ cell population generated more vmDA neurons and exhibited less proliferative potential (i.e., fewer Ki67^+^ cells) than unsorted or TPBG^−^ cell populations at up to 16 weeks after transplantation.

## Discussion

Despite three decades of experimental and clinical work, ethical and logistical concerns surrounding fetal brain tissue prevent human fetal mesencephalic tissue from being a clinically competitive option for PD treatment. Since hPSCs emerged as a promising cell source for transplantation over a decade ago^27^, multiple protocols for the differentiation of hPSCs into vmDA neurons have been reported^10–14^.

Our differentiation method was based on four signaling molecules that were previously reported to synergistically induce hPSC neuralization and ventralization. Dorsomorphin (DM) and SB431542 (SB) are inhibitors of bone morphogenetic protein and activin/nodal signaling, respectively, and were used first for neuroectodermal induction^23^. CHIR99021 [a glycogen synthase kinase-3 (GSK-3) inhibitor] and SAG [a Sonic hedgehog (SHH) signaling agonist] were subsequently used for regional specification and ventralization^10^. Fibroblast growth factor 8 (FGF8)—which is implicated in midbrain and hindbrain specification—was added in some protocols^10,13,18,28^, but not others^11,29,30^. In our experiments, vmDA specification was efficiently induced without adding FGF8, leading us to omit FGF8 from the protocol.

A critical issue for successful cell transplantation is the homogeneity of the cell population. Enhanced homogeneity is advantageous for reducing potential contamination of residual undifferentiated hPSCs and other unwanted cells, which could lead to tumor formation. FACS of fluorescent reporter cells, such as LMX1A-eGFP knock-in cells^25,31^, can be used to enrich LMX1A^+^ vmDA precursors. However, genetically modified cells must be carefully examined for the presence of off-target mutations in order to be used for clinical applications. In that sense, it would be a safe and convenient approach to sort the cells using cell surface markers expressed specifically in LMX1A^+^ vmDA precursors.

To this end, using LMX1A-eGFP knock-in cell lines, we identified several cell surface markers (ALCAM, CD47, CORIN, and TPBG) that were preferentially expressed in LMX1A^+^ cells relative to LMX1A^−^ cells. ALCAM, CD47, and CORIN were previously reported as potential vmDA neuron-related cell surface markers^13,16,19^. ALCAM is a central nervous system microvascular endothelium marker. ALCAM is expressed in the broad floor-plate area containing the midbrain and more caudal regions during early embryonic development^22^. Therefore, cells obtained using anti-ALCAM antibodies contain many cell types other than vmDA precursors. CD47, an integrin-associated protein, is also reported as a cell surface marker for the enrichment of vmDA precursors via targeting FOXA2^+^ cells^19^. However, as most cells in the unsorted population derived by our differentiation protocol expressed FOXA2 ( ≥ 83%), and CD47 was expressed in more than 95% of total cells on D20, CD47 might not be a suitable marker for the enrichment of vmDA precursors using our protocol. Consistent with our results, a previous study reported that a CD47 antibody is not able to enrich FOXA2^+^ vmDA precursors^21^. CORIN, a floor-plate marker, was shown to be broadly expressed in caudal floor plate areas and ventral mesencephalon during early development^13,16^. Only a small population of cells (12.4%) was obtained by MACS targeting CORIN on D20 after differentiation, suggesting that CORIN-targeted sorting was not an appropriate option for our differentiation protocol.

TPBG, also known as trophoblast glycoprotein, 5T4, or WNT-activated inhibitory factor 1 (WAIF1), is expressed in trophoblasts and several carcinomas^32–35^. TPBG is also highly expressed in LMX1A^+^ cells relative to LMX1A^−^ cells. There are no reports of TPBG expression in the ventral midbrain or the role of TPBG in vmDA neuronal development and function. Our *in silico* analysis using the data generated by La Manno and colleagues^36^ revealed that TPBG is expressed in medial floor-plate progenitors, lateral floor-plate progenitors, and DA2-type neurons. Intriguingly, DA2-type neurons differentiate into DA^1A^-type neurons in the substantia nigra of the ventral midbrain^26,37^.

TPBG-based cell sorting enriched LMX1A^+^FOXA2^+^ vmDA precursors, with LMX1A^+^FOXA2^+^ double-positive cells comprising 41.1% and 54.2% of unsorted and TPBG-sorted populations, respectively. The TPBG^+^ group also generated significantly more TH^+^ (64.8%), NURR1^+^ (56.6%), and PITX3^+^ (23.6%) cells in vitro compared with the unsorted (TH^+^: 45.3%, NURR1^+^: 39.3%, and PITX3^+^: 14.6%) and TPBG^−^ groups (TH^+^: 5.67%, NURR1^+^: 6.4%, and PITX3^+^: 0.9%) (Fig. 4).

After transplantation into a rodent PD model, more TH^+^, NURR1^+^, and PITX3^+^ neurons were found in the grafts of TPBG^+^ cells, compared with those in TPBG^−^ or unsorted cells. The percentage of TH^+^ cells in the TPBG^+^ group was approximately 35.82%, while those in the unsorted group and TPBG^−^ group were 20.24% and 3.02%, respectively (Fig. 5e). Intriguingly, more GIRK2^+^ cells were detected in the TPBG^+^ group than in the TPBG^–^ group. These data are consistent with the increased RNA expression of *SOX6*, a marker of A9 DA precursors, in the TPBG^+^ group (Fig. 3d). These results indicate that TPBG-targeted cell sorting enriched authentic vmDA precursors, which became A9 vmDA neurons after transplantation. In addition, fewer Ki67^+^ cells were apparent in the TPBG^+^ cell grafts than in the TPBG^–^ and unsorted cell grafts. These results indicate that sorting vmDA precursors with an anti-TPBG antibody reduced unwanted cell types, such as those with persistent proliferative capability in vivo.

Because the percentage of TH^+^ cells in TPBG^+^ grafts was approximately 35.82%, it would be of interest to know the identity of the rest of the cells. We detected a substantial number of TUJ1^+^ cells (neurons) and GFAP^+^ cells (astrocytes) in the TH^−^hNCAM^+^ cell population (Supplementary Fig. 12a and b). This result somewhat agrees with a previous report claiming that the majority of the TH^−^hNCAM^+^ cells in the grafts were TUJ1^+^^10^. Significantly, serotonergic (5-HT^+^) neurons, the cause of graft-induced dyskinesia^38^, were hardly detected in any of the groups derived using our differentiation protocol (Supplementary Fig. 13a). Furthermore, only a few glutamatergic neurons (0.68%) were detected in our study (Supplementary Fig. 13b).

Transplantation of TPBG^+^ cells efficiently ameliorated behavioral dysfunction in a rodent PD model. TPBG^+^ cells contained more vmDA precursors (LMX1A^+^FOXA2^+^ double-positive cells) than unsorted cells (54.2% vs. 39.1%, respectively; Fig. 2c) and produced higher percentages of TH^+^ cells in the graft than unsorted cells (35.8% vs. 20.2%, respectively; Fig. 5e). However, no significant differences in functional recovery were observed between the TPBG^+^ and unsorted groups up to 16 weeks after transplantation. This result may be explained by a previous observation that only ~1,000 vmDA neurons are sufficient for behavioral recovery^39^. Because we transplanted 350,000 cells of each cell population (i.e., TPBG^+^ cells and unsorted cells), it is possible that no difference in functional recovery could be detected between the two cell populations. Therefore, behavioral assessment after transplantation with smaller numbers of cells would provide a clearer answer on the functional benefits of TPBG-sorted cells.

In summary, we identified a novel surface marker, TPBG, that could enrich A9 vmDA precursors for possible cell therapy for PD. Our study can aid in further strengthening the efficacy and safety of hPSC-mediated cell replacement therapy for PD.

## Methods

### Ethical approval

All experimental procedures were approved by the Institutional Animal Care and Use Committees of Yonsei University Health System (2016-0088, 2019-0087).

### Human embryonic stem cell culture and differentiation

Undifferentiated hESCs [H9 (WA09); WiCell Inc., USA] and hESC reporter lines were cultured on a layer of mitomycin C (Sigma-Aldrich, St. Louis, MO, USA)-treated STO feeder cells (American Type Culture Collection) in Dulbecco’s Modified Eagle Medium (DMEM)/F12 (Thermo Fisher Scientific, Waltham, MA, USA) supplemented with 20% knockout-serum replacement (KSR; Thermo Fisher Scientific), 1× nonessential amino acid (Thermo Fisher Scientific), 0.1 mM 2-mercaptoethanol (Sigma-Aldrich), and 4 ng/ml of basic fibroblast growth factor (bFGF; R&D System, Minneapolis, MN, USA). The cells were passaged weekly onto fresh feeder cells by mechanical passaging. hESC colonies were detached with 2 mg/ml type IV collagenase (Worthington Biochemical Corp., Lakewood, NJ, USA) and embryoid bodies (EBs) were generated by incubating the colony fragments in EB medium (hESC culture medium without bFGF) containing 1.5% dimethyl sulfoxide for the first 24 h. The EBs were cultured in suspension for an additional four days in EB medium supplemented with 5 µM dorsomorphin (Merck, Kenilworth, NJ, USA) and 5 μM SB431542 (Sigma-Aldrich). On day 5, EBs were attached to Matrigel-coated culture dishes and cultured for six days in bmN2 medium (DMEM/F12 medium containing 1 × N2 supplement, and 20 ng/ml bFGF, 20 µg/ml human insulin solution) supplemented with 1 µM CHIR99021 (Miltenyi Biotec, Bergisch Gladbach, Germany) and 0.5 µM Smoothened Agonist (SAG; Merck). Distinct neural rosette structures at this stage were mechanically isolated and replated on Matrigel-coated dishes for 2-day expansion in bN2B27 medium (DMEM/F12 containing 1 × N2, 1 × B27, and 20 ng/ml bFGF) supplemented with 1 µM CHIR99021 and 0.5 µM SAG. On day 13 after differentiation, cell clusters containing vmDA precursors were dissociated into single cells with Accutase (Merck) and replated on Matrigel-coated plates in N2B27 medium (bN2B27 medium without bFGF). Replated vmDA precursors were grown in N2B27 medium for another seven days until 90% confluency was reached. To differentiate vmDA precursors into mature DA neurons, vmDA precursors were cultured in NBG medium [DMEM/F12, 1 × N2, 0.5 × B27, 0.5 × G21 supplement (Gemini Bio-Products, West Sacramento, CA, USA)] supplemented with 1 µM of DAPT (Sigma-Aldrich) for seven days. Cells were then further differentiated for about three weeks in NBG medium supplemented with 10 ng/ml brain-derived neurotrophic factor (BDNF; ProSpec-Tany TechnoGene, Israel), 10 ng/ml glial cell-derived neurotrophic factor (GNDF; ProSpec-Tany TechnoGene), 200 µM ascorbic acid (Sigma-Aldrich), and 1 µM db-cAMP (Sigma-Aldrich).

### Generation of the LMX1A-eGFP reporter hESC line

The LMX1A-eGFP targeting vector was constructed using pUC19 as the plasmid backbone with the following layout: 5′ homology arm-endogenous *LMX1A* genomic fragment (left arm)-T2A-eGFP-bGH poly(A)-PGK promoter-driven puromycin-resistance cassette-bGH poly(A)-3′ homology arm (right arm). Transcription activator-like effector nuclease (TALEN) sites were designed to cause double-strand breaks near the stop codon, TGA, in exon 9 of the *LMX1A* gene: 5′-TCC ATG CAG AAT TCT TAC TT-3′ (left) and 5′-TCA CAG AAC TCT AGG GGA AG-3′ (right). In total 1.0 × 10^7^ cells/ml of single cell-dissociated hESCs were mixed with 6 µg of TALEN-encoding plasmids (3 µg of each plasmid) and donor DNA plasmid and pulsed at 850 V for 30 ms (Neon transfection system; Thermo Fisher Scientific, Waltham, MA, USA). Starting at day 5 post electroporation, antibiotic selection was performed using 0.5 µg/ml puromycin. Puromycin-resistant colonies were individually picked and further grown for 10–14 days after electroporation.

### Genotyping of the LMX1A-eGFP hESC reporter line

Genomic DNA (gDNA) was extracted using a DNeasy Blood & Tissue Kit (QIAGEN, Hilden, Germany) according to the manufacturer’s instructions. gDNA polymerase chain reaction (PCR) was performed with the following parameters using the primers listed in Supplementary Table 2: initial denaturation at 95 °C for 30 sec, 35 cycles of denaturation at 95 °C for 10 s, annealing at 60 °C for 30 s, extension at 72 °C for 1 min, and final extension at 72 °C for 10 min.

### Fluorescence-activated cell sorting (FACS)

Isolation of eGFP-expressing cells from vmDA precursors derived from the LMX1A-eGFP hESC reporter line was initiated D20. Expanded cells were exposed to 10 μM of ROCK inhibitor for 1 h to prevent cell death. Cells were harvested and gently dissociated into single cells using Accutase and strained through a 40-µm cell strainer. Dissociated cells were resuspended in Sorting Buffer supplemented with 3% fetal bovine serum (FBS) and 1× penicillin-streptomycin in Hanks’ balanced salt solution at a final concentration of 2 × 10^6^ cells per ml. FACS was performed using a BD FACSAria-III cell sorter and FACSDiva software (BD Biosciences, San Jose, CA, USA) with the sorting strategies shown in Supplementary Fig. 14. Using a 488-nm laser for excitation, the eGFP^+^ and eGFP^−^ fractions were determined according to fluorescence intensity in the eGFP channel (505-nm long-pass, 535/30-nm band-pass filters). A fraction of cells was passed through the cell sorter, without selection, forming the unsorted group, which was then used as a control.

### Immunocytochemistry and flow cytometry

Cells were fixed in 4% paraformaldehyde (PFA) in phosphate-buffered saline (PBS) solution. For visualization of intracellular markers, cells were permeabilized with 0.1% Triton X-100-PBS solution, blocked with 2% bovine serum albumin (BSA)-PBS solution for 1 h at room temperature (RT), and incubated overnight at 4 °C with primary antibodies (Supplementary Table 3). Appropriate fluorescence-tagged secondary antibodies were used for visualization. Images were obtained using an Olympus IX71 microscope equipped with a DP71 digital camera, Olympus FSX100 system, or LSM710 confocal microscope (Carl Zeiss, Oberkochen, Germany). For flow cytometry, single cell-dissociated cells were fixed in 4% PFA-PBS solution. For detection of intracellular markers, cells were permeabilized with 1× Perm/Wash buffer (BD Biosciences) and incubated in 2% BSA-PBS solution with primary antibodies for 1 h at RT. Fluorescence-tagged secondary antibodies were used for visualization. Flow cytometry was performed using LSRII (BD Biosciences), and analysis was performed using FlowJo software (FlowJo, Ashland, OR, USA).

### Gene expression analysis and transcriptome profiling

Total RNA was isolated using the Easy-Spin Total RNA Extraction kit (iNtRON Biotechnology, South Korea). Complementary DNA was synthesized from 1 µg of total RNA using PrimeScript RT Master Mix (TAKARA Bio Inc., Kusatsu, Shiga, Japan). mRNA levels were quantified by quantitative reverse transcription-PCR (qRT-PCR) assays using SYBR Premix Ex Taq (TAKARA Bio Inc.) and CFX96 Real-Time System (Bio-Rad, Hercules, CA, USA). For each targeted gene, cycle-threshold (Ct) values were normalized to those of GAPDH. Normalized expression levels of targeted genes were compared among sorted, unsorted, and control samples based on the comparative Ct method. Data are expressed as the mean relative expression ± standard error of the mean (SEM) of at least three independent experiments. Sequences of primers used for gene expression analysis are listed in Supplementary Table 4. For microarray analysis, 10 µg of total RNA from each sample was processed and analyzed by Macrogen Inc. (Seoul, South Korea), and samples were hybridized to the Affymetrix Human U133 Plus 2.0 array. Transcriptome analyses were performed in triplicate. Genes that were up- or downregulated in eGFP^+^ (LMX1A^+^) cells compared with eGFP^−^ (LMX1A^−^) cells with an adjusted *p*-value <0.05 and fold change ≥2 were considered statistically significant.

### Magnetic-activated cell sorting (MACS)

To isolate cell surface marker-positive cells via MACS, expanded cells at D20 were dissociated into single cells as described above. Dissociated cells were stained with primary antibodies (Supplementary Table 3) in 1% FBS-PBS solution for 30 min at 4 °C, and labeled cells were incubated with 20 µl Microbeads (Miltenyi Biotec) per 1 × 10^7^ total cells for 30 min. Marker-positive cells were separated from marker-negative cells using a separation column (LS column; Miltenyi Biotec) following the manufacturer’s instructions. Sorted cells were replated on Matrigel-coated culture dishes in N2B27 medium. After two days, in vitro characterization and in vivo transplantation were performed.

### Analysis of dopamine release

Fully matured cells (i.e., eight weeks after terminal differentiation) were used in this analysis. Cells were washed with a low KCl solution (2.5 mM CaCl_2_, 11 mM glucose, 20 mM HEPES-NaOH 4.7 mM KCl, 1.2 mM KH_2_PO_4_, 1.2 mM MgSO_4_, and 140 mM NaCl) and incubated in low KCl solution at 37°C for 2 min. The solution was subsequently replaced with a high KCl solution (2.5 mM CaCl_2_, 11 mM glucose, 20 mM HEPES-NaOH, 60 mM KCl, 1.2 mM KH_2_PO_4_, 1.2 mM MgSO_4_, and 85 mM NaCl) and cells were further incubated at 37°C for 15 min. The solution was collected in 15 ml tubes and centrifuged for 1 min at 2000 rpm to remove the debris. The supernatant was collected in 1.5-ml tubes and stored at −80°C before use. Dopamine concentration was measured using a dopamine ELISA kit (Cat. No. KA3838; Abnova, Taipei, Taiwan) according to the manufacturer’s instructions.

### In vivo transplantation into a 6-hydroxydopamine (6-OHDA) rodent PD model and immunohistochemistry

Adult female Sprague-Dawley rats weighing 200–250 g were used for transplantation (Orient Bio Inc., South Korea). For anesthesia, 30 mg/kg Zoletil (Virbac, Carros, France) was combined with 10 mg/kg Rompun (Bayer, Leverkusen, Germany). Rats were made hemi-parkinsonian by injecting 3 µl of 30 mM 6-OHDA into the medial forebrain bundle using the following coordinates: TB −0.45, AP −0.40, ML −0.13, and DV −0.70. At least four weeks post lesion, 4 µl of 8.75 × 10^4^ cells/µl cell suspension (total of 350,000 cells) were stereotaxically transplanted into each rat at the following coordinates: TB −0.24, AP +0.08, ML −0.30, and DV −0.40/−0.50. Daily intraperitoneal injections of immunosuppressive cyclosporine A (10 mg/kg) were administered beginning two days before transplantation and continuing every day thereafter until the rats were sacrificed. Sixteen weeks after transplantation, the rats were anesthetized and transcardially perfused with 0.9% saline solution followed by 4% PFA. Cryoprotected brains were embedded in FSC 22 compound, and 18-µm serial coronal sections were sliced using a cryostat. Progressive hematoxylin and eosin staining was performed. For immunofluorescence staining, brain tissue sections were incubated with the appropriate primary antibodies overnight after blocking in 3% BSA-PBS solution containing 0.3% Triton X-100. Fluorescent secondary antibodies were used for visualization. 3,3′-diaminobenzidine (DAB) staining was performed using the ABC HRP kit (Cat. No. PK-6101, VECTOR Laboratories, Burlingame, CA, USA) and DAB substrate kit (Cat. No. C09-12, GBI Labs, Bothell, WA, USA) according to the manufacturer’s instructions. The antibodies used are listed in Supplementary Table 3.

### Graft quantifications

To estimate dopamine neurons within the graft, coronal sections of striatal brain regions (five per brain) were scanned using an Aperio AT2 (Leica Biosystems, Wetzlar, Germany) under 20× magnification. The number of DAB-stained TH^+^ cells were counted manually with Image J software (v1.53c, NIH, Bethesda, MD, USA). The final counts were corrected for series number (1:24) to estimate the total number of TH^+^ cells per animal. The DA yield is presented as the number of TH^+^ cells per 100,000 transplanted cells. For graft volume quantification, the coronal sections of striatal brain areas were scanned using an Aperio AT2 under 20× magnification and analyzed using ImageScope software (Leica Biosystems, v12.3.0.506). The graft area was extrapolated in every section of the 1:24 series that showed human neural cell adhesion molecule 1 (hNCAM^+^) staining, and the graft volumes were calculated using Cavalieri’s principle. As hNCAM labels both nuclei and processes, only the densest core of the grafts was included in the measurements. The graft volume was normalized to 100,000 transplanted cells to enable comparison between experiments. The number of mouse brains that were transplanted with the following cells: unsorted cells, *n* = 5; TPBG^+^ cells, *n* = 4; TPBG^−^ cells, *n* = 3.

### Rodent behavioral analysis

The amphetamine-induced rotation test was performed 30 min after the intraperitoneal injection of 2.5 mg/kg amphetamine (Sigma-Aldrich). Rotation tests were also performed before transplantation, and at 4, 8, 12, and 16 weeks after transplantation.

### Statistical analysis

Data are shown as the mean ± SEM or standard deviation (SD) of at least three independent experiments. Data were analyzed using paired or unpaired two-tailed Student’s *t*-tests or analysis of variance (ANOVA) when two or more groups were involved. *P*-values <0.05 were considered statistically significant.

### Reporting summary

Further information on research design is available in the Nature Research Reporting Summary linked to this article.

## Supplementary information

Supplementary Information

Reporting Summary

## Data Availability

All data generated or analyzed during this study are included in this published article (and its supplementary information files). The microarray data that support the findings of this study have been deposited in GEO with the accession code GSE171769.
