# Supplementary material for: Trophoblast glycoprotein is a marker for efficient sorting of ventral mesencephalic dopaminergic precursors derived from human pluripotent stem cells
Source: NPJ Parkinsons Dis. 2021 Jul 19;7:61. doi: 10.1038/s41531-021-00204-8 (PMC8289854; doi:10.1038/s41531-021-00204-8)
Supplement: Supplementary file 1 — Supplementary Information [file 41531_2021_204_MOESM1_ESM.pdf]

Supplementary Information

# Trophoblast glycoprotein is a marker for efficient sorting of ventral mesencephalic dopaminergic precursors derived from human pluripotent stem cells

Jeong-Eun Yoo, Dongjin R. Lee, Sanghyun Park, Hye-Rim Shin, Kun Gu Lee, Dae-Sung Kim, Mi-Young Jo, Jang-Hyeon Eom, Myung Soo Cho, Dong-Youn Hwang, and Dong-Wook Kim

## Supplementary Fig. 1

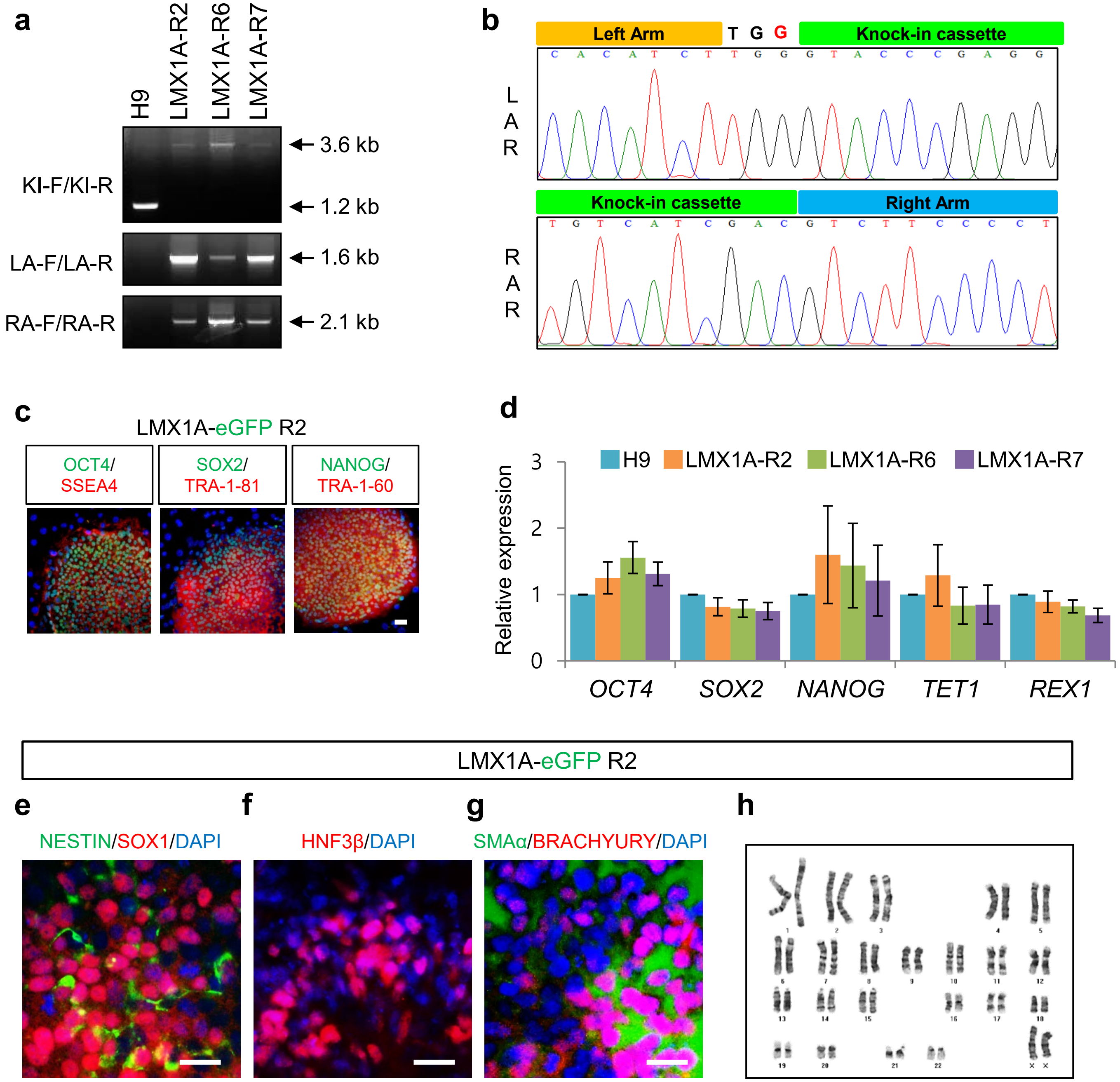

**Supplementary Fig. 1. Characterization of the LMX1A-eGFP reporter hESC line. a** PCR-based integration analysis produced a band of 3.6 kb (*upper bands in the top panel*) from the reporter clones and 1.2 kb (*lower band in the top panel*) from H9 (WT) hESCs with KI-F and KI-R primers (Supplementary Table 2). The LMX1A-eGFP reporter hESC lines (LMX1A-R2, LMX1A-R6, and LMX1A-R7) each had a biallelic insertion of eGFP at the *LMX1A* locus. Other bands (*middle and bottom panels*) were only detected at the knock-in *LMX1A* locus. Bands of 1.6 kb with LA-F and LA-R primers and 2.1 kb with RA-F and RA-R primers were detected by PCR-based integration analysis of LMX1A-eGFP reporter hESC lines. **b** DNA sequencing analysis revealed insertion of the eGFP gene into exon 9 of the *LMX1A* locus with a nonstop mutation (TGA > TGG). **c** The LMX1A-eGFP reporter hESC lines exhibited hESC-like morphology and expressed pluripotent stem cell markers OCT4 (green), SOX2 (green), NANOG (green), SSEA4 (red), TRA-1-81 (red), and TRA-1-60 (red). DAPI (blue) was used as a counterstain. **d** qRT-PCR revealed similar mRNA levels of the pluripotency-associated genes *OCT4*, *SOX2*, *NANOG*, *TET1*, and *REX1* between LMX1A-eGFP hESC reporter lines and parental hESCs (H9). Data are shown as mean ± SEM of three independent experiments. **e**-**g** LMX1A-eGFP reporter hESC lines underwent *in vitro* differentiation. With differentiation cues, the reporter hESC line differentiated into each of the three germ layers: ectoderm [NESTIN (green), SOX1(red)], endoderm [HNF3β (red)], and mesoderm [SMAα (green), BRACHYURY (red)]. DAPI staining was used to determine total cell content. **h** LMX1A-eGFP reporter hESC line had a normal karyotype. Scale bars, 50 μm (**c**) and 25 μm (**e**-**g**). hESC, human embryonic stem cell; KI, knock-in.

# Supplementary Fig. 2

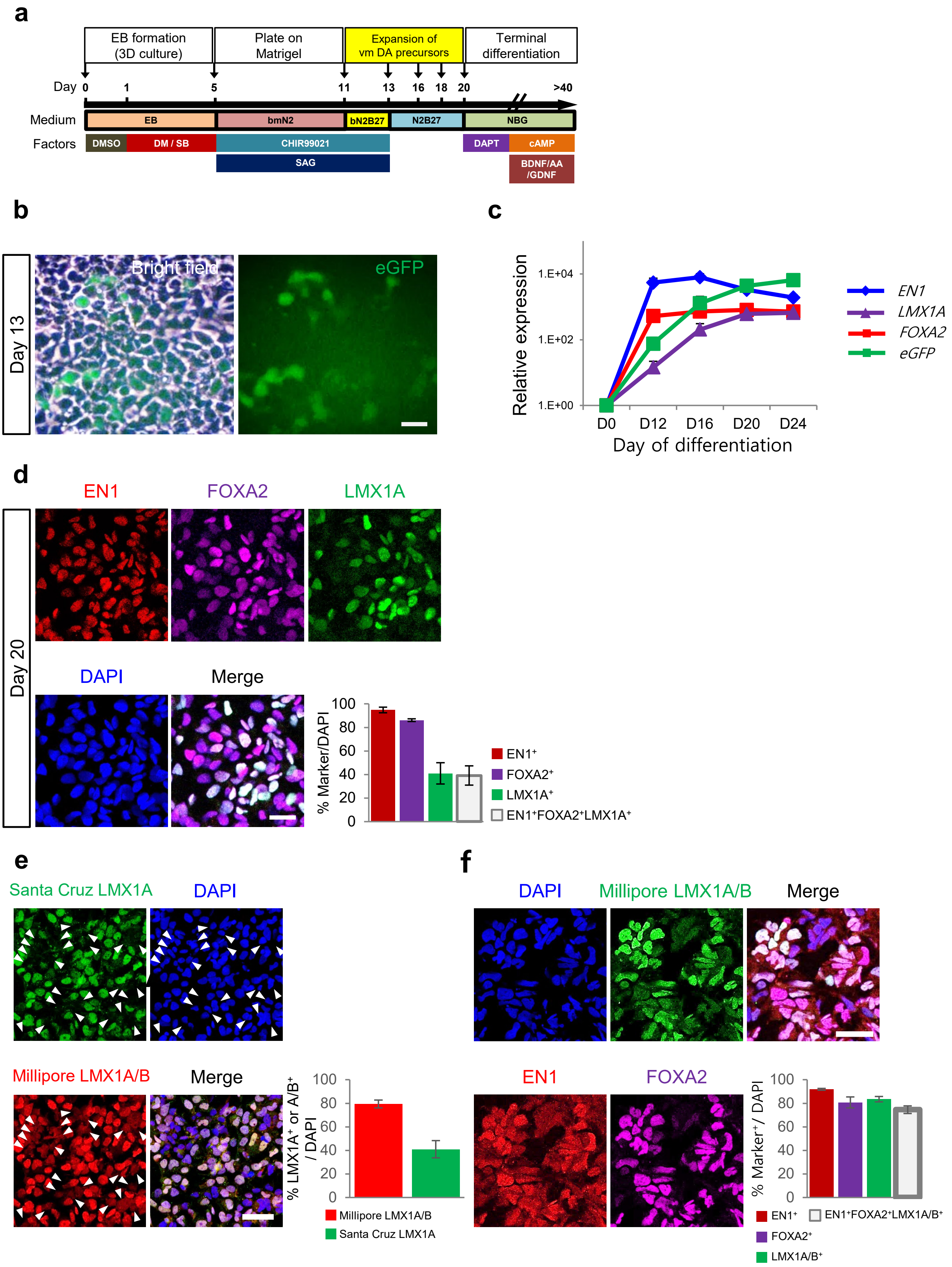

**Supplementary Fig. 2. Differentiation of LMX1A-eGFP reporter hESCs into vmDA neurons.** **a** Schematic overview of the stages involved in the differentiation protocol. **b** eGFP expression in cultures derived from LMX1A-eGFP hESCs on D13. **c** Relative expression of eGFP and vmDA regional identity markers (i.e., *EN1*, *FOXA2*, and *LMX1A*) determined by qRT-PCR during differentiation of LMX1A-eGFP reporter hESCs relative to undifferentiated H9 hESCs (D0). Data are shown as mean ± SEM from three biological replicates. **d** EN1 (red), FOXA2 (magenta), and LMX1A (green) expression in vmDA precursors on D20. Whole-cell populations were detected using DAPI (blue). The numbers of $EN1^+$, $FOXA2^+$, $LMX1A^+$, and $EN1^+FOXA2^+LMX1A^+$ cells were counted. Data are shown as mean ± SD of three biological replicates. **e** Immunofluorescence staining with a Santa Cruz LMX1A antibody (green), and a Millipore LMX1A/B antibody (red). DAPI (blue) staining was used to determine total cell content. White arrowheads indicate cells that were detected by Millipore LMX1A/B antibody, but not by Santa Cruz LMX1A antibody (*left panels*). The percentages of $LMX1A^+$ and $LMX1A/B^+$ cells across the total cell populations are shown (*right panel*). Data are shown as mean ± SD of three biological replicates. **f** EN1 (red), FOXA2 (magenta), and LMX1A/B (Millipore antibody) (green) expression in vmDA precursors on D20. Whole-cell populations were detected using DAPI (blue). The numbers of $EN1^+$, $FOXA2^+$, $LMX1A/B^+$, and $EN1^+FOXA2^+LMX1A/B^+$ cells were counted. Data are shown as mean ± SD of three biological replicates. Scale bars, 25 μm (**b, d**), 50 μm (**e**-**f**). AA, ascorbic acid; BDNF, brain-derived neurotrophic factor; cAMP, dibutyryl-cyclic AMP; DAPT, γ-secretase inhibitor IX; DM, dorsomorphin; DMSO, dimethyl sulfoxide; GDNF, glial cell-derived neurotrophic factor; SB, SB431542.

# Supplementary Fig. 3

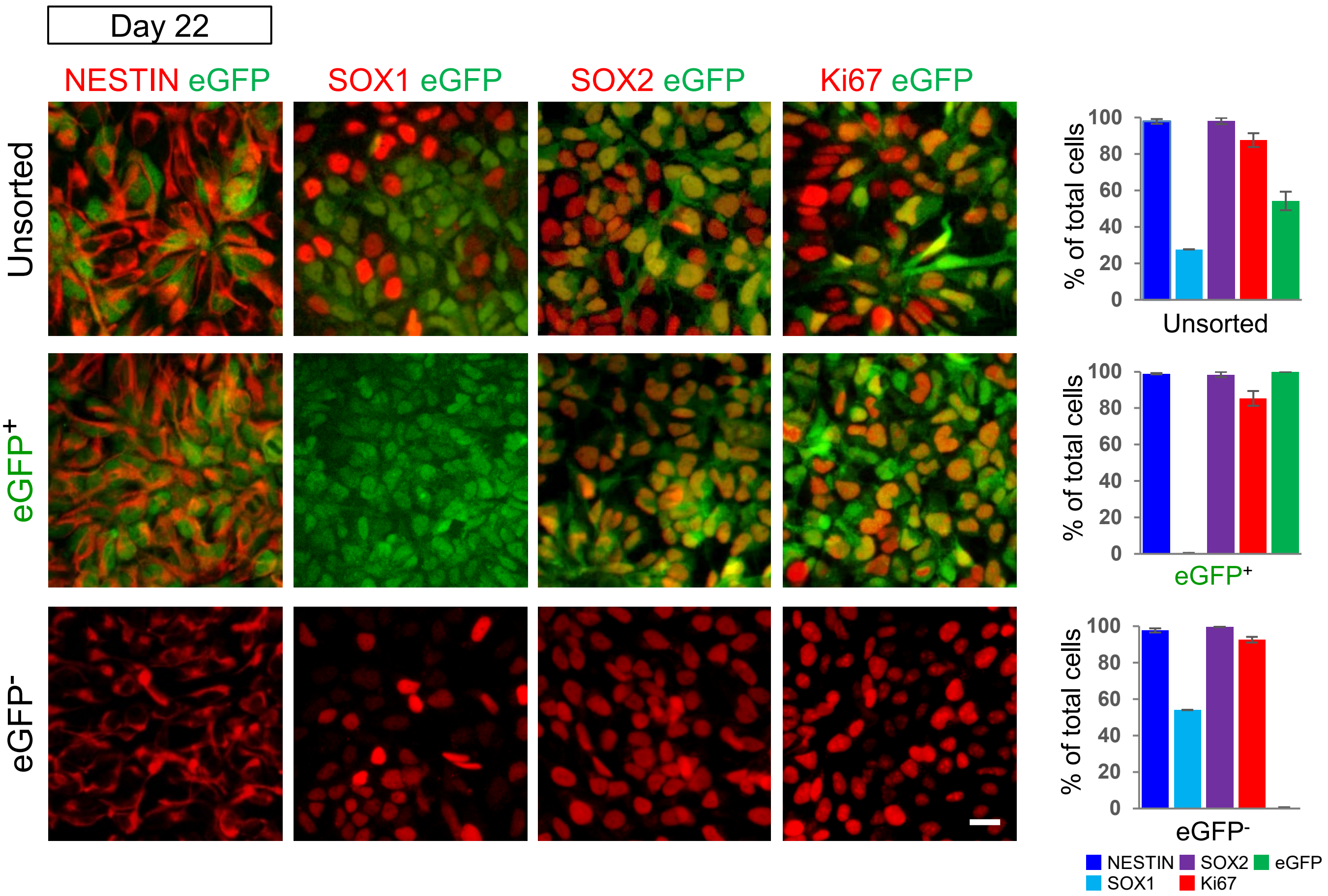


**Supplementary Fig. 3. Characterization of eGFP⁺ vmDA precursors.**

Immunofluorescence staining of eGFP (green), NESTIN (red), SOX1 (red), SOX2 (red), and Ki67 (red) in unsorted, eGFP⁺, and eGFP⁻ cells (*left panels*) at 2 days after FACS (D22). NESTIN⁺, SOX1⁺, SOX2⁺, Ki67⁺, and eGFP⁺ cells were quantified (*right panel*). Data are shown as mean ± SD of three independent experiments. Scale bar, 25 μm.

## Supplementary Fig. 4

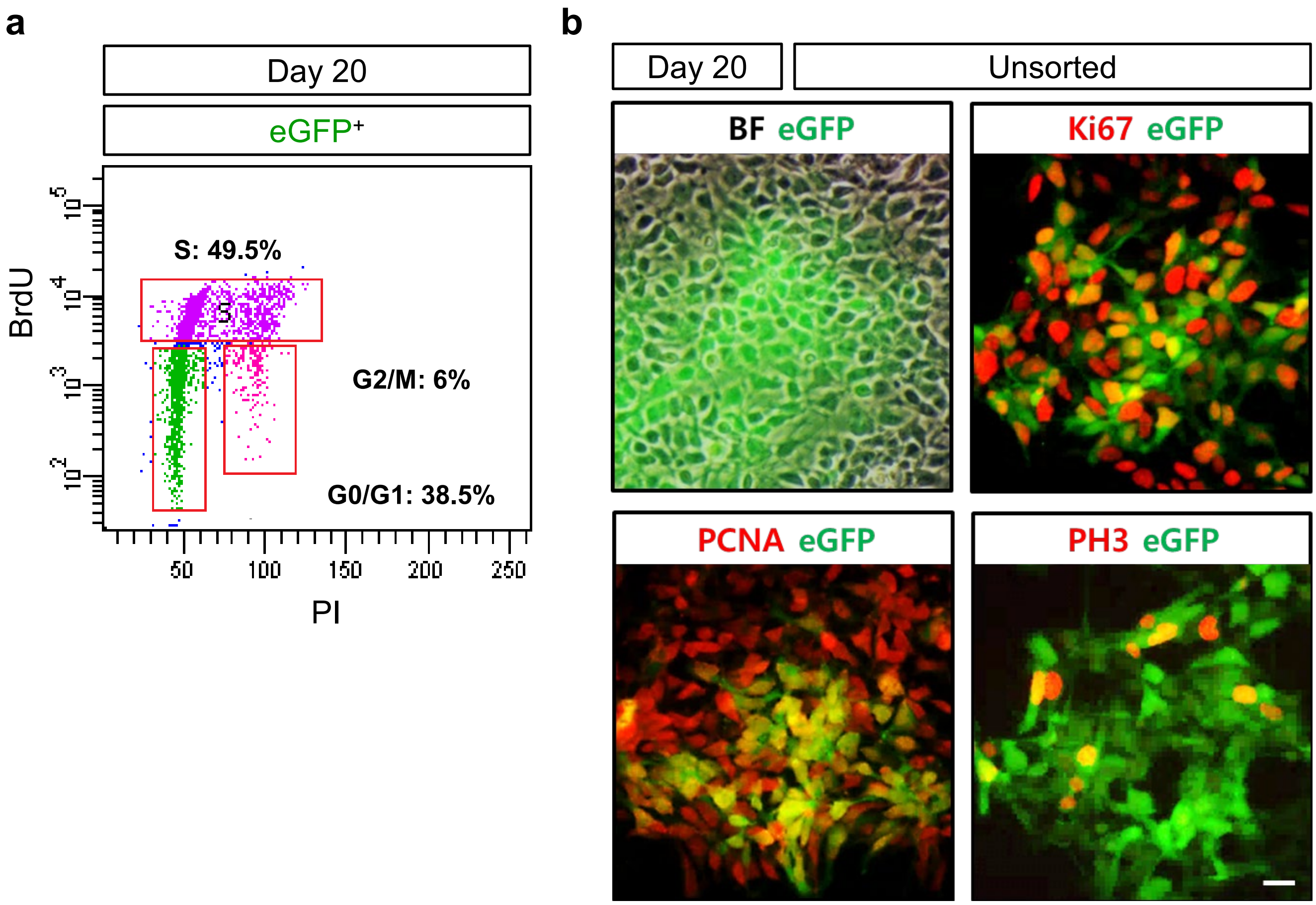


**Supplementary Fig. 4. Cell cycle analysis of eGFP⁺ vmDA precursors. a** Cell cycle analysis of eGFP⁺ cells at D20. **b** Representative bright-field (BF) and immunofluorescence images of vmDA precursors (D20) expressing eGFP (green), Ki67 (red), PCNA (red), and PH3 (red). Scale bar, 25 µm. BrdU, bromodeoxyuridine; PI, propidium iodide.

# Supplementary Fig. 5

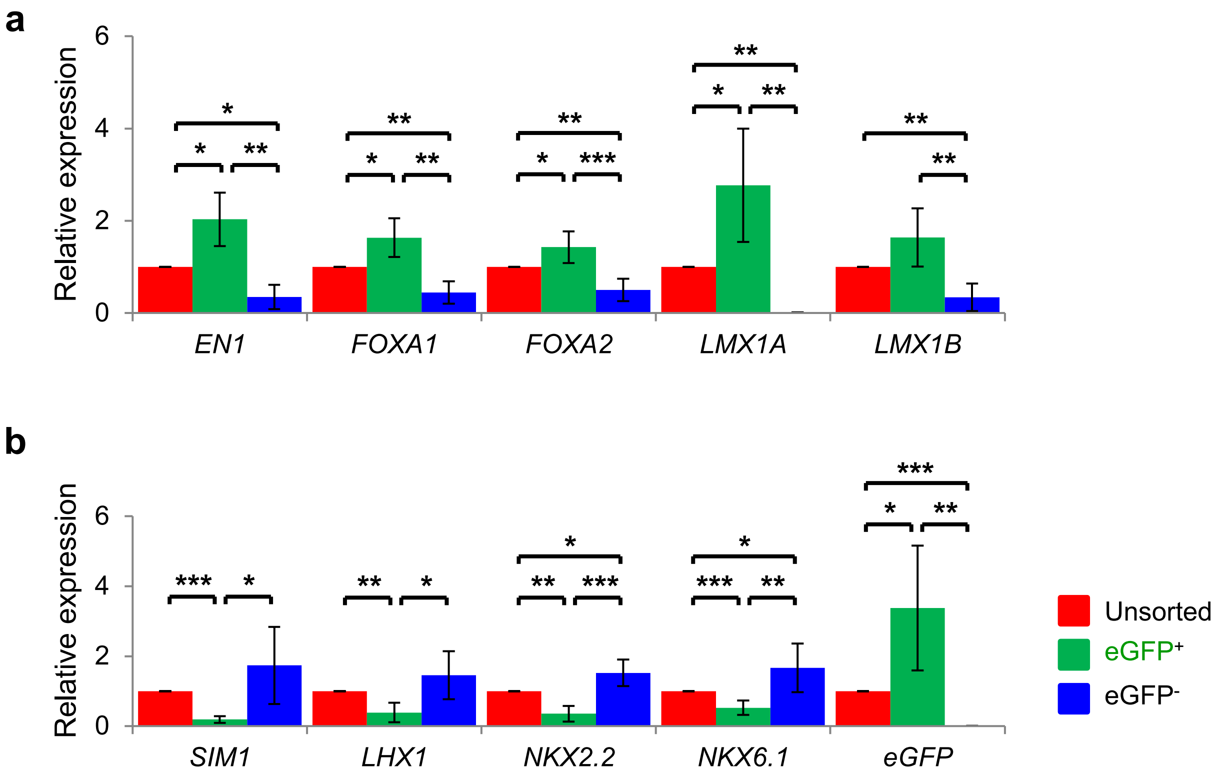


**Supplementary Fig. 5. qRT-PCR analysis of eGFP⁺ vmDA precursors. a** qRT-PCR analysis of relative expression of vmDA lineage and regional identity markers in unsorted, eGFP⁺, and eGFP⁻ cells on D20. Values for unsorted cells were arbitrarily set to 1. **b** Relative expression of a serotonergic precursor marker (*NKX 2.2*) and red nucleus progenitor markers (*SIM1*, *LHX1*, and *NKX6.1*) in unsorted, eGFP⁺, and eGFP⁻ cells on D20. Expression level of each gene in unsorted cells was arbitrarily set to 1. Data are shown as mean ± SEM of four biological replicates. *$p < 0.05$, **$p < 0.01$, ***$p < 0.001$, unpaired Student's *t*-tests.

## Supplementary Fig. 6

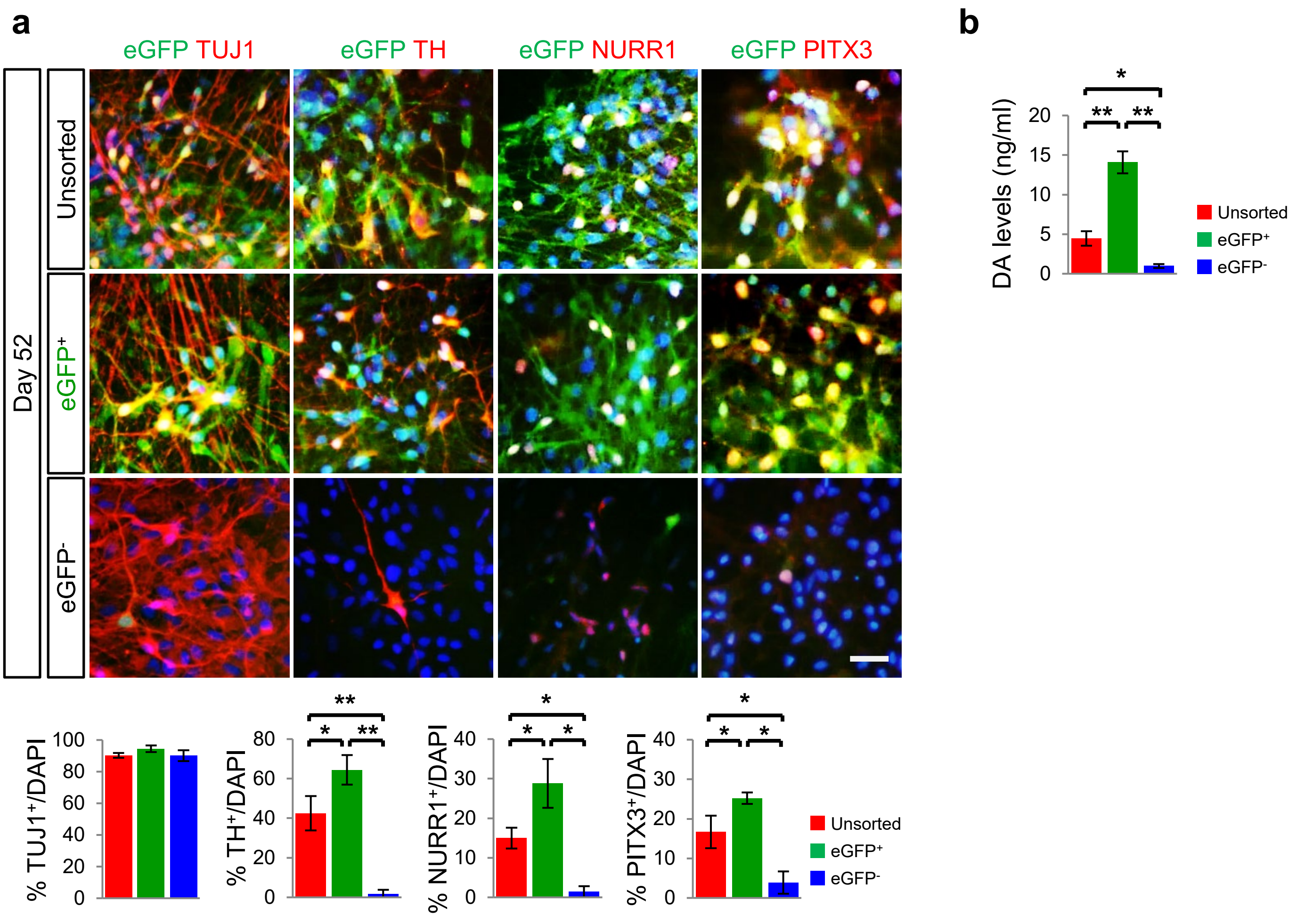


**Supplementary Fig. 6.** ***in vitro*** **differentiation of LMX1A⁺ vmDA precursors. a** Immunofluorescence staining for eGFP (green), TUBB3 (red), TH (red), NURR1 (red), and PITX3 (red) in terminally differentiated unsorted, eGFP⁺, and eGFP⁻ cells on D52. DAPI (blue) staining was used to determine total cell content (*top panels*). Quantification of TUBB3⁺, TH⁺, NURR1⁺, and PITX3⁺ cells (*bottom panel*). Data are shown as mean ± SD of three biological replicates. **b** Dopamine levels in culture media of LMX1A-eGFP hESC reporter line-derived unsorted, eGFP⁺, or eGFP⁻ cells after terminal differentiation for approximately 8 weeks. Data are shown as mean ± SD of three biological replicates. *$p < 0.05$, **$p < 0.01$, one-way ANOVA with Bonferroni's multiple comparison tests. Scale bars, 25 μm. DA, dopamine.

# Supplementary Fig. 7

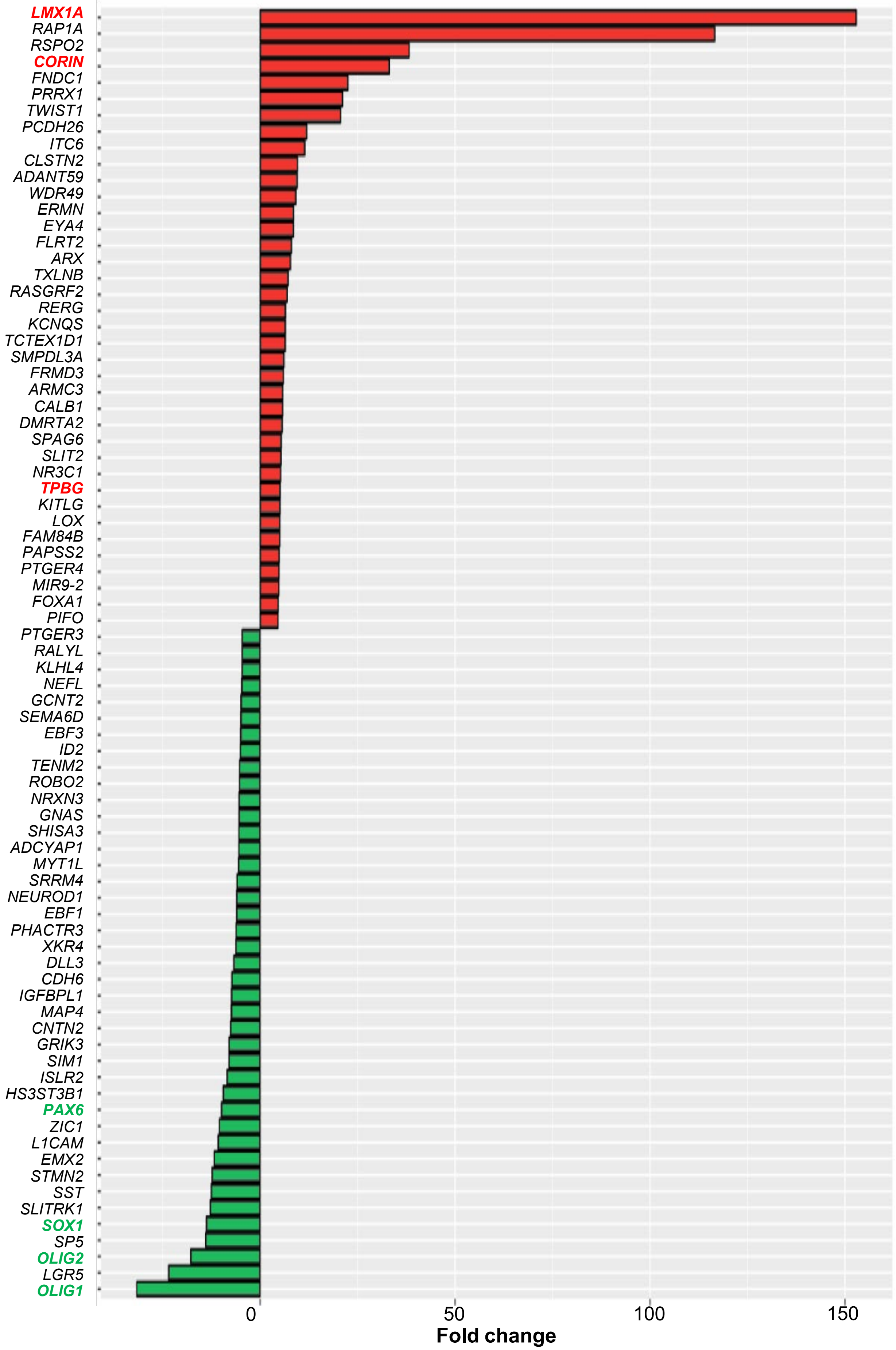

**Supplementary Fig. 7. List of 78 differentially expressed genes.** Upregulated (red) and downregulated (green) genes in $eGFP^+$ cells ($LMX1A^+$ cells) relative to $eGFP^-$ cells ($LMX1A^-$ cells). Transcriptome analyses were performed in triplicate. The genes that were mentioned in the text were denoted in red or green color.

# Supplementary Fig. 8

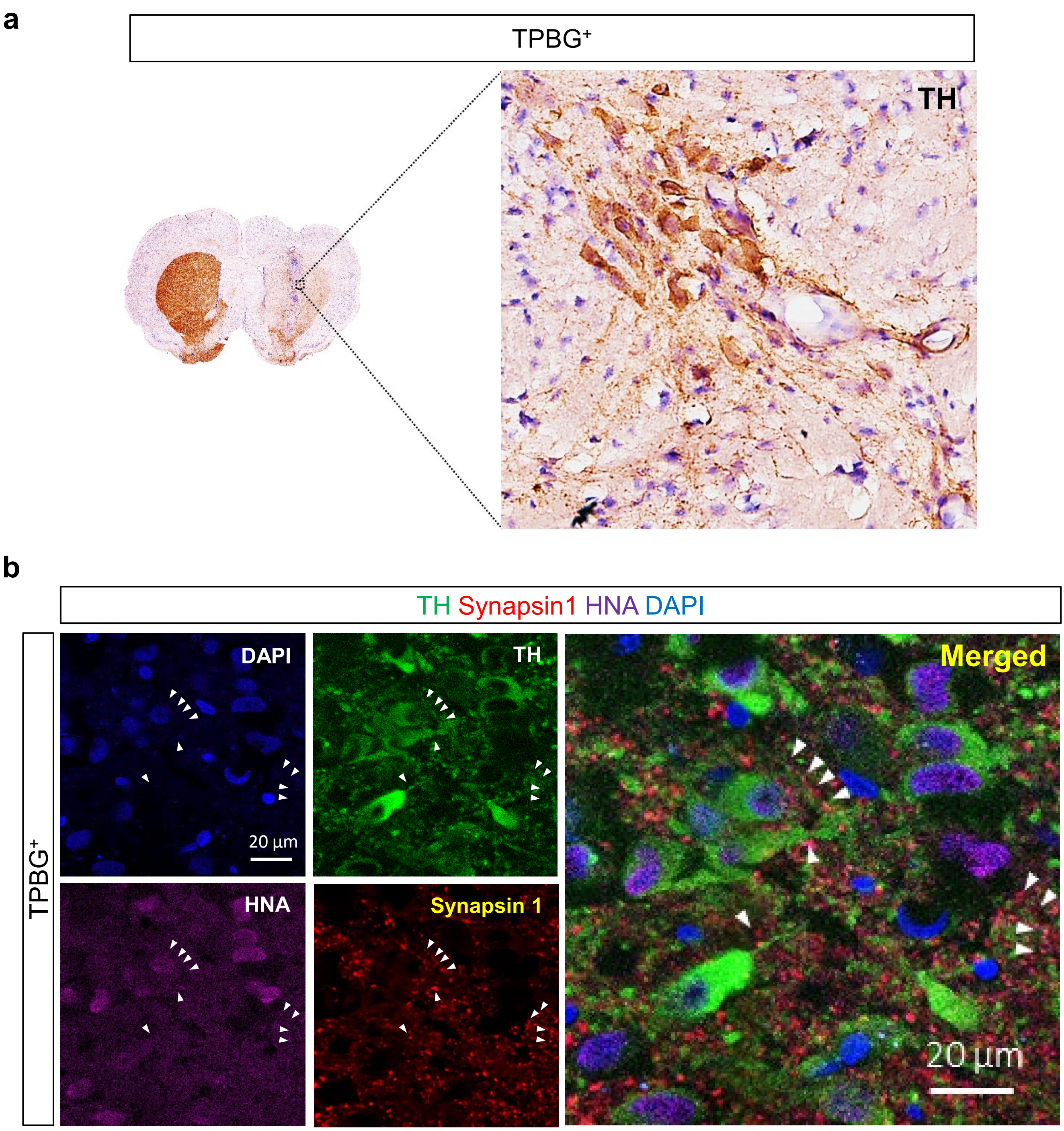


**Supplementary Fig. 8. Engrafted TH⁺ cell innervation and integration. a** Immunostaining with TH evidently showed many processes derived from the engrafted vmDA neurons in the striatum. **b** Punctate signals (white arrowheads) of synapsin 1 colocalized with signals from the engrafted TH⁺ (HNA⁺TH⁺) neurons, indicating striatal innervation of the engrafted DA neurons. Scale bar, 20 μm.

# Supplementary Fig. 9

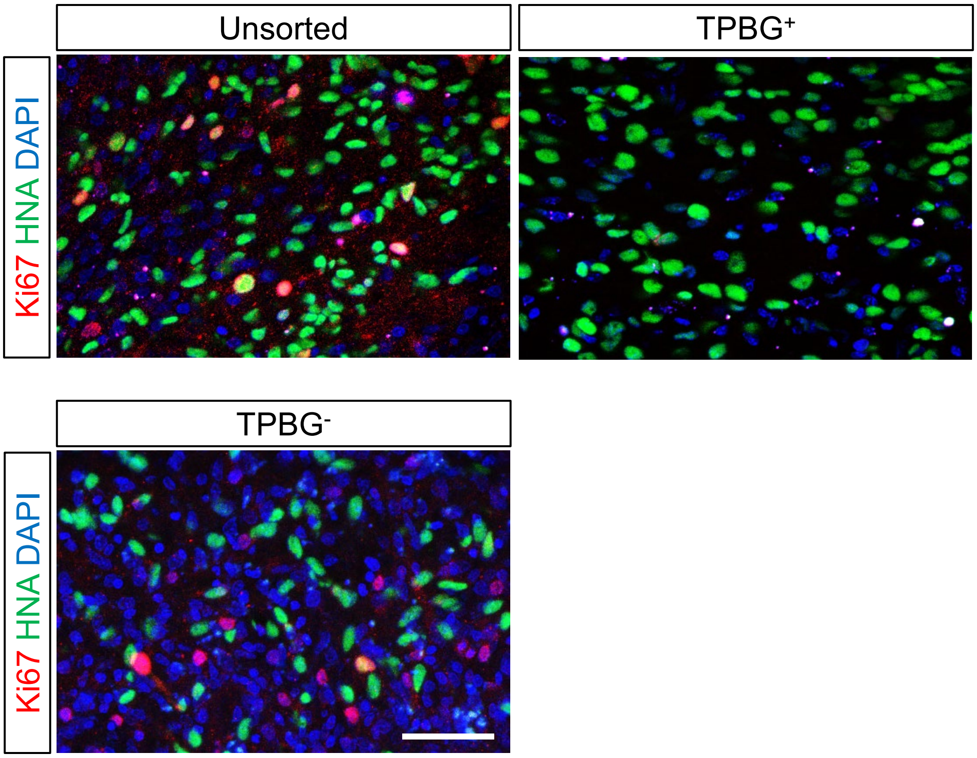


**Supplementary Fig. 9. Growth and survival of TPBG$^+$ vmDA precursors *in vivo*.**

Immunofluorescence staining of HNA (green), Ki67 (red) in grafts containing unsorted cells, TPBG$^+$ cells, and TPBG$^-$ cells at 16 weeks after transplantation. DAPI (blue) was used as a counterstain. Scale bar, 50 μm.

## Supplementary Fig. 10

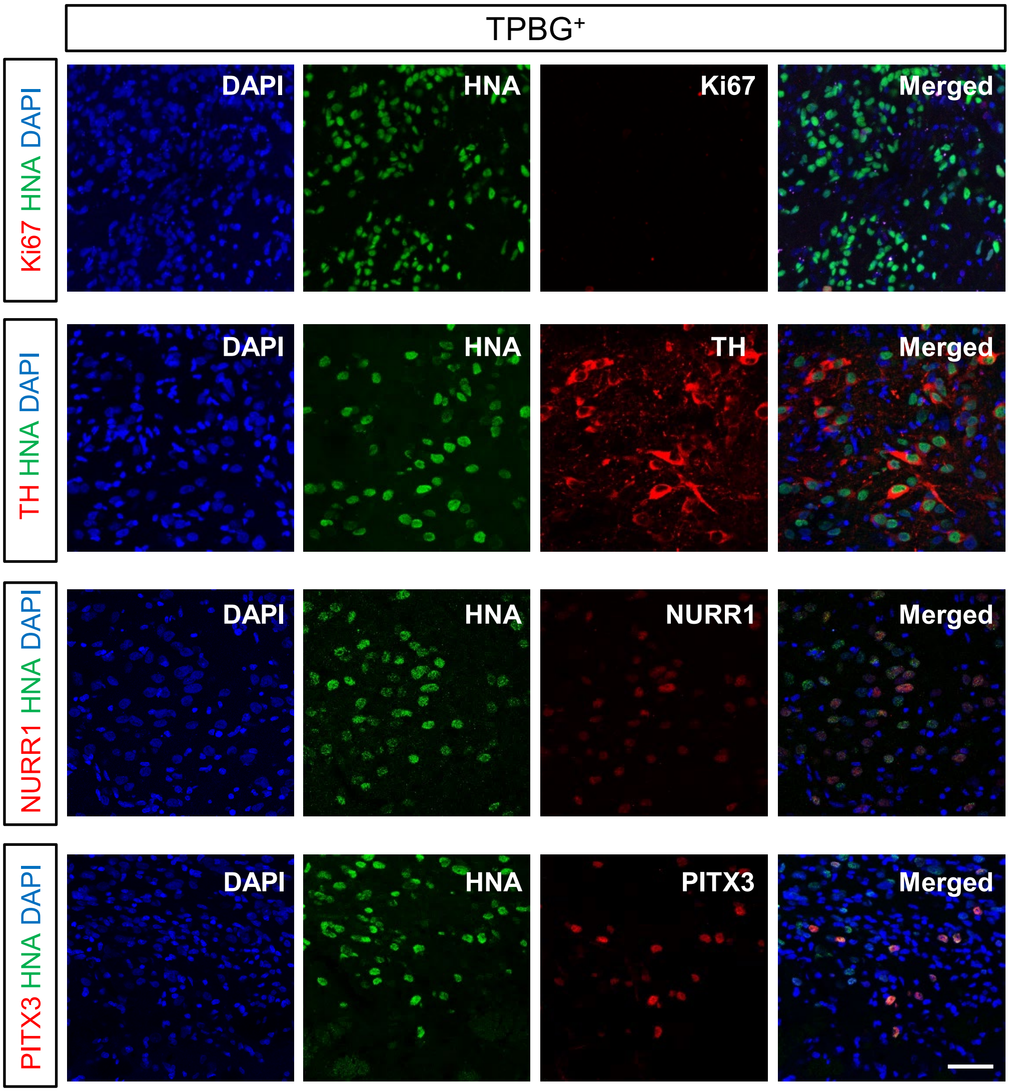


**Supplementary Fig. 10. Characterization of TPBG+ grafts with HNA immunofluorescence staining.** Expression of HNA (green), Ki67 (red), TH (red), NURR1 (red), and PITX3 (red) in grafts containing TPBG+ cells at 16 weeks after transplantation. DAPI (blue) was used as a counterstain. Scale bar, 50 μm.

# Supplementary Fig. 11

**a**

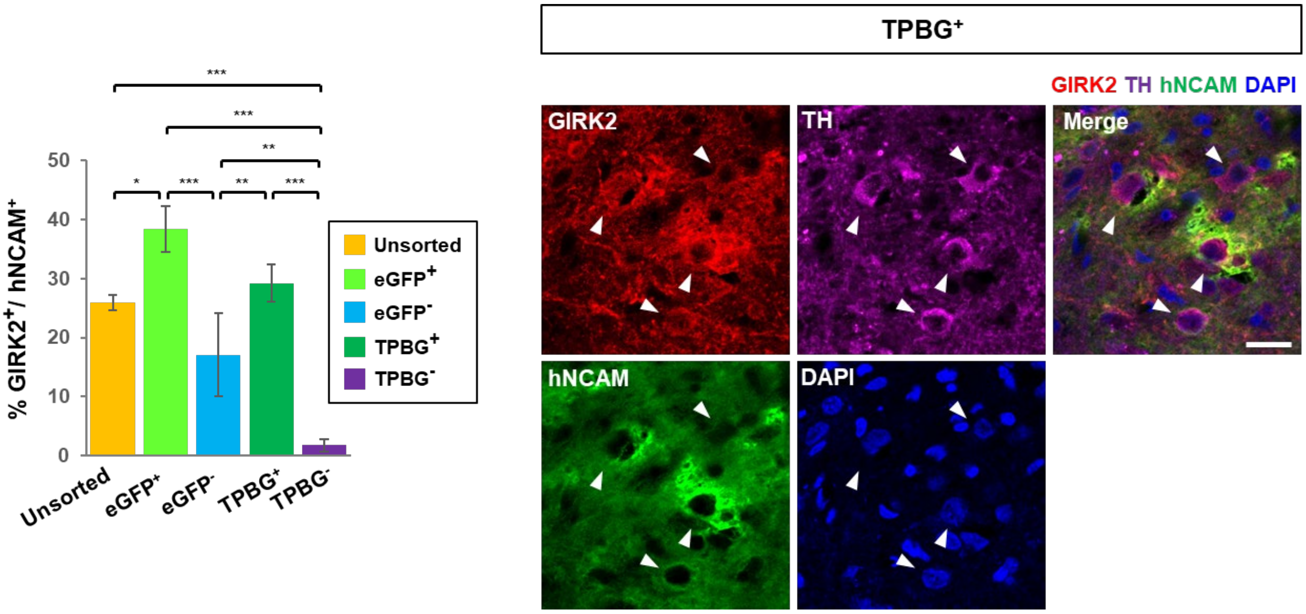


**b**

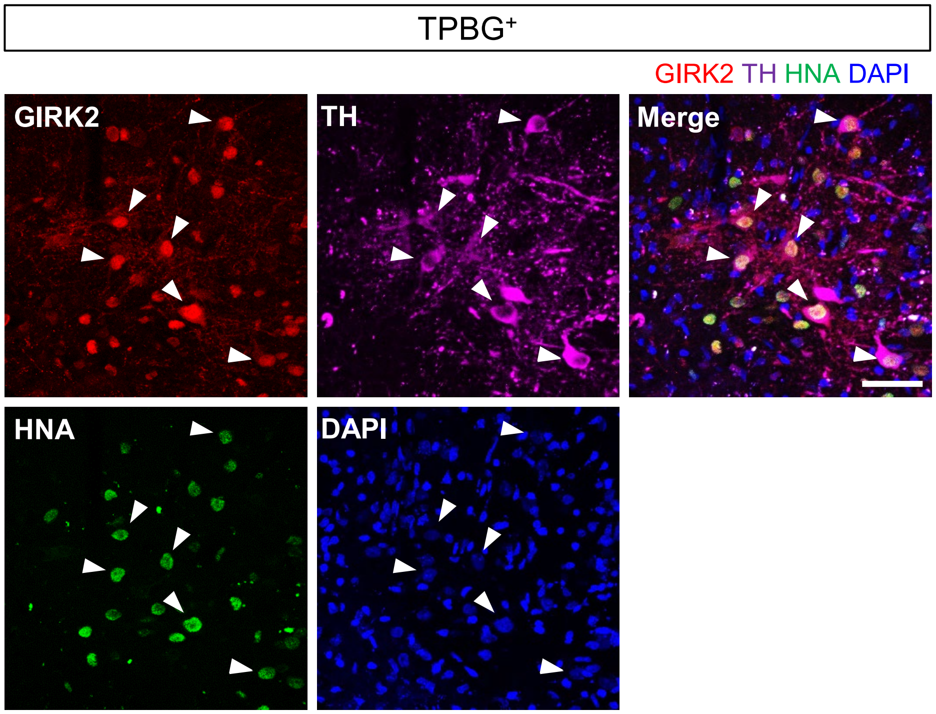

**Supplementary Fig. 11. A9 vmDA marker expression in the grafts. a** Quantification of GIRK2$^+$ cells in grafts containing unsorted cells (n = 3), eGFP$^+$ cells (LMX1A$^+$ cells; n = 3), eGFP$^-$ cells (LMX1A$^-$ cells; n = 4), TPBG$^+$ cells (n = 4), and TPBG$^-$ cells (n = 4) at 16 weeks after transplantation. *$p < 0.05$, ** $p < 0.01$, *** $p < 0.001$, one-way ANOVA with Bonferroni's multiple comparison tests. The graft containing TPBG$^+$ cells expressed hNCAM (green), GIRK2 (red), and TH (magenta). DAPI (blue) staining was used to determine total cell content. **b** The graft containing TPBG$^+$ cells was examined for expression of GIRK2 (red), TH (magenta) , and the human-specific nuclear marker HNA (green). DAPI (blue) staining was used to determine total cell content. Scale bars, 20 μm (**a**), 50 μm (**b**).

# Supplementary Fig. 12

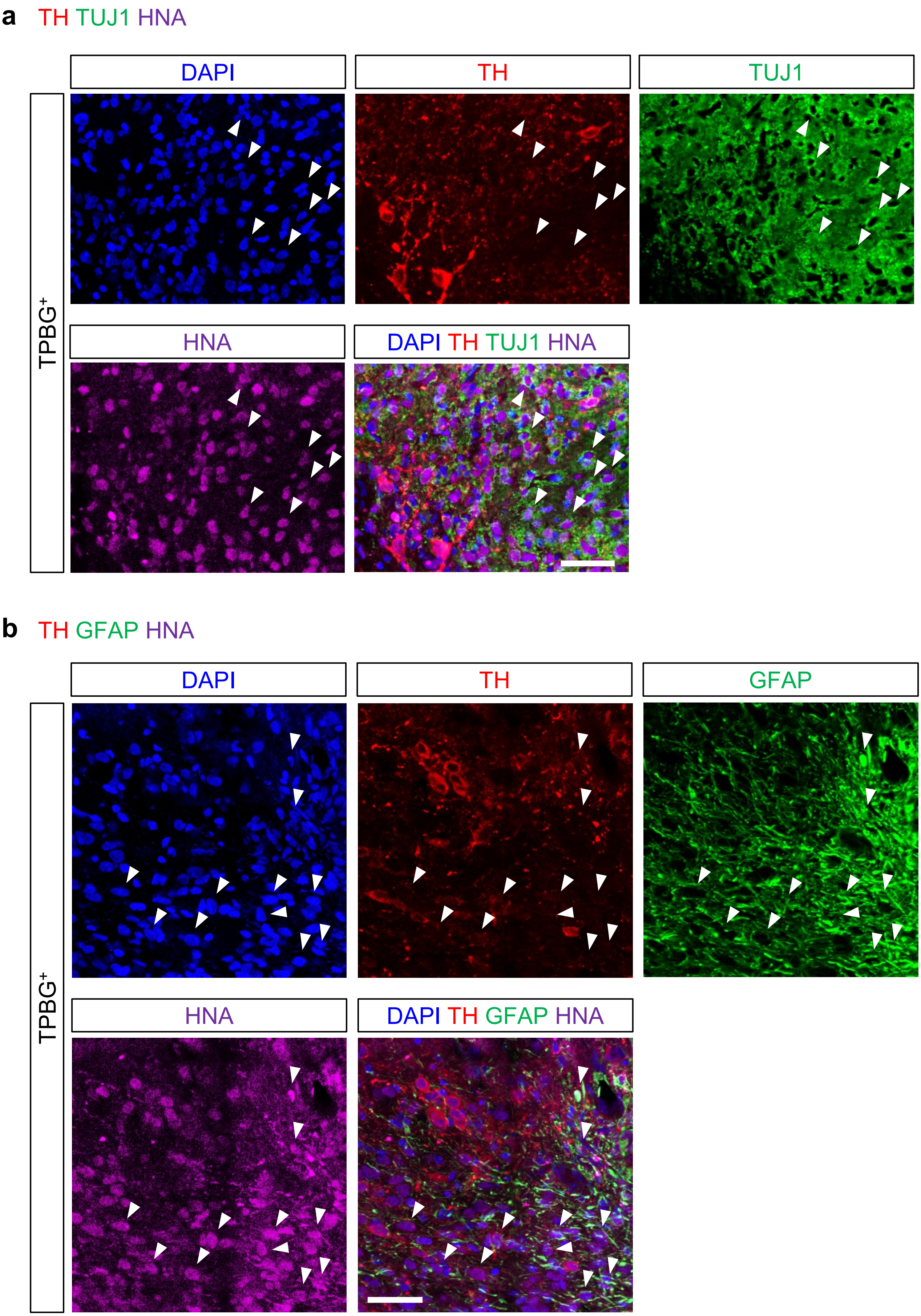

**Supplementary Fig. 12. Characterization of non-DA cells in TPBG$^+$ grafts. a** Immunofluorescence images of TPBG$^+$ grafts expressing TUJ1 (green), TH (red), and HNA (magenta). DAPI (blue) staining was used to determine total cell content. White arrowheads indicate TH$^-$TUJ1$^+$HNA$^+$ cells. **b** Immunofluorescence images of TPBG$^+$ grafts expressing GFAP (green), TH (red), and HNA (magenta). DAPI (blue) staining was used to determine total cell content. White arrowheads indicate glial lineage cells derived from transplanted cells (TH$^-$GFAP$^+$HNA$^+$ cells). Scale bars, 50 μm.

## Supplementary Fig. 13

**a**

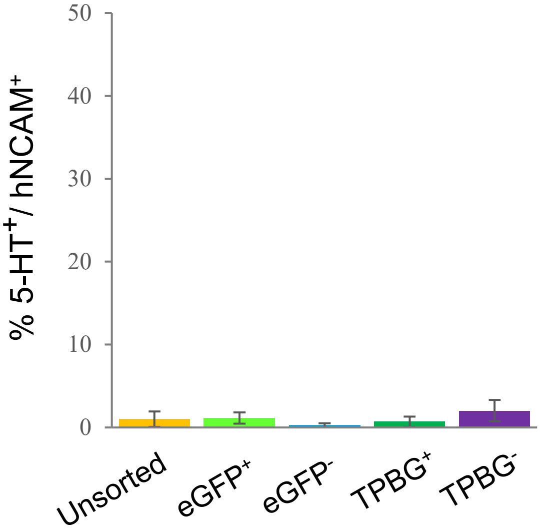


**b**

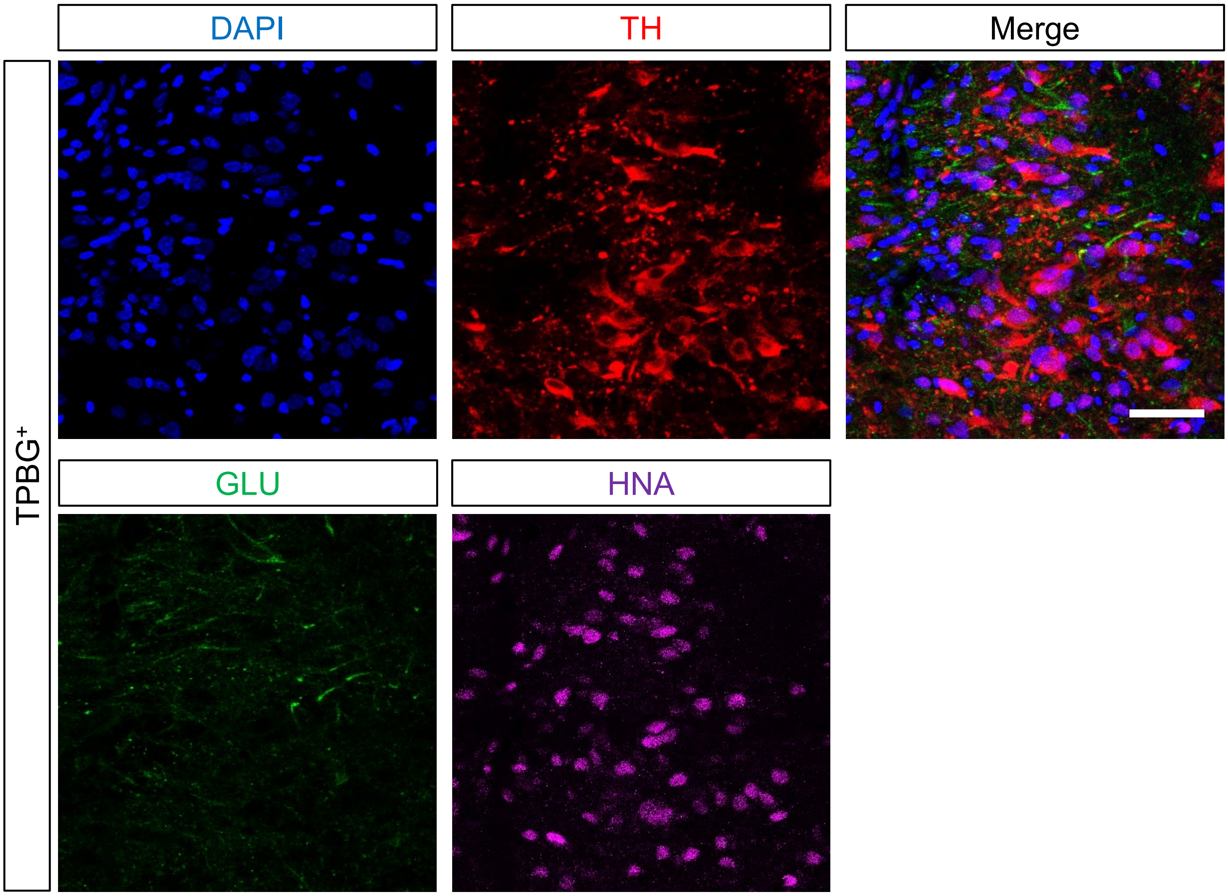

**Supplementary Fig. 13. Non-DA neuron characterization in $TPBG^{+}$ grafts. a** $5\text{-}HT^{+}$ cells were counted in grafts containing unsorted cells (n = 3), $eGFP^{+}$ cells ($LMX1A^{+}$ cells; n = 3), $eGFP^{-}$ cells ($LMX1A^{-}$ cells; n = 4), $TPBG^{+}$ cells (n = 5), and $TPBG^{-}$ cells (n = 5) at 16 weeks. **b** Representative immunofluorescence images of $TPBG^{+}$ grafts expressing TH (red), GLU (green), and HNA (magenta). DAPI (blue) was used as a counterstain. Scale bar, 50 μm.

# Supplementary Fig. 14

**Negative Control**
H9-derived vmDA precursors (D20)

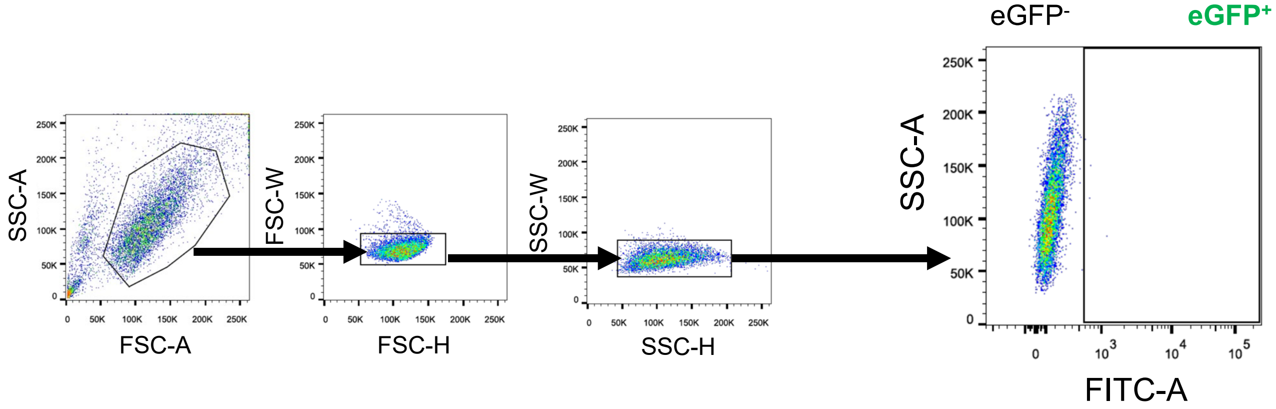


LMX1A-eGFP R2-derived vmDA precursors (D20)

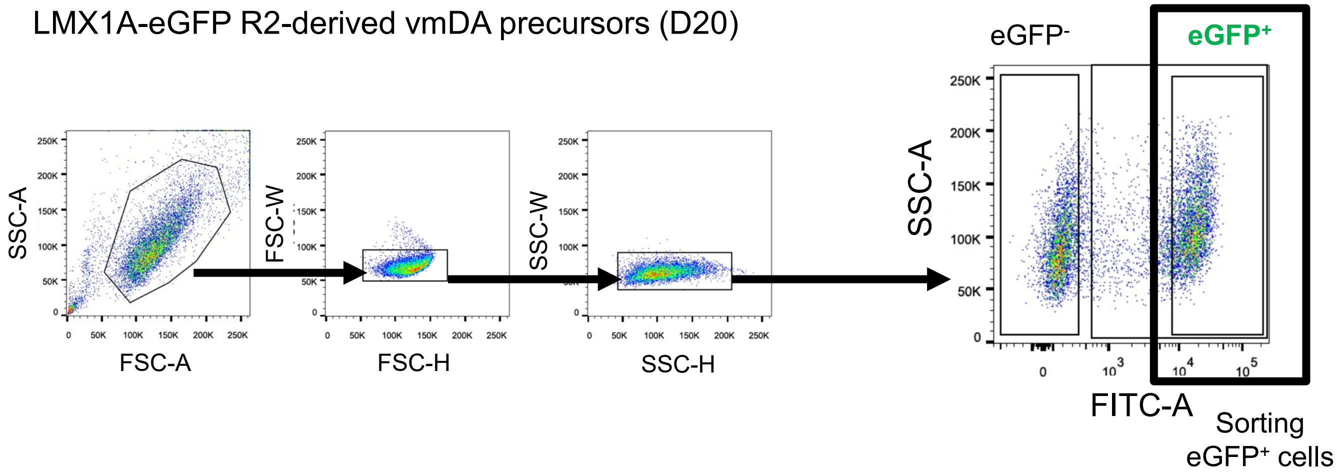


**Supplementary Fig. 14. FACS gating strategies for sorting eGFP$^{+}$ cells.**

Supplementary Table 1. Upregulated transmembrane genes in eGFP$^+$ cells compared with eGFP$^-$ cells.

| No | | Gene ID | Symbol | Name | Fold Change |
|---|---|---|---|---|---|
| 1 | | 10699 | ***CORIN*** | Corin | 33.10 |
| 2 | * | 64881 | *PCDH20* | Protocadherin 20 | 12.00 |
| 3 | * | 64084 | *CLSTN2* | Calsyntenin 2 | 9.56 |
| 4 | * | 23768 | *FLRT2* | Fibronectin leucine-rich repeat transmembrane protein 2 | 8.04 |
| 5 | | 56479 | *KCNQ5* | Potassium Voltage-Gated Channel Subfamily Q Member 5 | 6.46 |
| 6 | | 257019 | *FRMD3* | FERM Domain Containing 3 | 5.98 |
| 7 | | 9353 | *SLIT2* | Slit Guidance Ligand 2 | 5.36 |
| 8 | * | 7162 | ***TPBG*** | Trophoblast Glycoprotein (oncofetal antigen 5T4) | 5.14 |
| 9 | * | 4254 | *KITLG* | KIT Ligand (SCF) | 5.11 |
| 10 | | 5734 | *PTGER4* | Prostaglandin E Receptor 4 | 4.84 |
| 11 | * | 84898 | *PLXDC2* | Plexin Domain Containing 2 (TEM7R) | 4.01 |
| 12 | * | 388336 | *SHISA6* | Shisa Family Member 6 | 3.98 |
| 13 | | 65983 | *GRAMD3* | GRAM Domain Containing 2B | 3.89 |
| 14 | | 64078 | *SLC28A3* | Solute Carrier Family 28 Member 3 | 3.67 |
| 15 | * | 400451 | *FAM174B* | Family With Sequence Similarity 174, Member B | 3.55 |
| 16 | * | 92293 | *TMEM132C* | Transmembrane Protein 132C | 3.45 |
| 17 | | 83698 | *CALN1* | Calneuron 1 | 3.43 |
| 18 | | 400120 | *SERTM1* | Serine Rich And Transmembrane Domain Containing 1 | 3.40 |
| 19 | * | 961 | ***CD47*** | CD47 Molecule | 3.27 |
| 20 | * | 8324 | *FZD7* | Frizzled Class Receptor 7 | 3.22 |
| 21 | | 3708 | *ITPR1* | Inositol 1,4,5-Trisphosphate Receptor Type 1 | 3.21 |
| 22 | | 159371 | *SLC35G1* | Solute Carrier Family 35 Member G1 | 3.15 |
| 23 | * | 5099 | *PCDH7* | Protocadherin 7 | 3.13 |
| 24 | | 152189 | *CMTM8* | CKLF Like MARVEL Transmembrane Domain Containing 8 | 3.05 |
| 25 | | 2555 | *GABRA2* | Gamma-Aminobutyric Acid Type A Receptor Alpha2 Subunit | 3.00 |
| 26 | | 256987 | *SERINC5* | Serine Incorporator 5 | 2.86 |
| 27 | | 222894 | *FERD3L* | Fer3 Like BHLH Transcription Factor | 2.85 |
| 28 | | 57205 | *ATP10D* | ATPase Phospholipid Transporting 10D | 2.78 |
| 29 | * | 50937 | *CDON* | Cell Adhesion Associated, Oncogene Regulated (CDO) | 2.78 |
| 30 | | 148641 | *SLC35F3* | Solute Carrier Family 35 Member F3 | 2.77 |
| 31 | | 3755 | *KCNG1* | Potassium Voltage-Gated Channel Modifier Subfamily G Member 1 | 2.74 |
| 32 | | 158326 | *FREM1* | FRAS1 Related Extracellular Matrix 1 | 2.64 |
| 33 | * | 3672 | *ITGA1* | Integrin Subunit Alpha 1 | 2.62 |
| 34 | | 51454 | *GULP1* | GULP PTB Domain Containing Engulfment Adaptor 1 | 2.61 |
| 35 | | 203859 | *ANO5* | Anoctamin 5 | 2.58 |
| 36 | | 57628 | *DPP10* | Dipeptidyl Peptidase Like 10 | 2.55 |
| 37 | | 389432 | *SAMD5* | Sterile Alpha Motif Domain Containing 5 | 2.52 |

(*Continued*)

| No | | Gene ID | Symbol | Name | Fold Change |
|---|---|---|---|---|---|
| 38 | | 25825 | *BACE2* | Beta-Secretase 2 | 2.51 |
| 39 | | 114990 | *VASN* | Vasorin | 2.47 |
| 40 | * | 214 | ***ALCAM*** | Activated Leukocyte Cell Adhesion Molecule(CD166) | 2.42 |
| 41 | | 64778 | *FNDC3B* | Fibronectin Type III Domain Containing 3B | 2.41 |
| 42 | | 2558 | *GABRA5* | Gamma-Aminobutyric Acid Type A Receptor Alpha5 Subunit | 2.38 |
| 43 | | 57537 | *SORCS2* | Sortilin Related VPS10 Domain Containing Receptor 2 | 2.27 |
| 44 | | 169200 | *TMEM64* | Transmembrane Protein 64 | 2.26 |
| 45 | * | 8321 | *FZD1* | Frizzled class receptor 1 | 2.26 |
| 46 | | 83464 | *APH1B* | Aph-1 Homolog B, Gamma-Secretase Subunit | 2.21 |
| 47 | | 157378 | *TMEM65* | Transmembrane Protein 65 | 2.14 |
| 48 | | 84976 | *DISP1* | Dispatched RND Transporter Family Member 1 | 2.13 |
| 49 | | 2891 | *GRIA2* | Glutamate Ionotropic Receptor AMPA Type Subunit 2 | 2.10 |
| 50 | | 51088 | *KLHL5* | Kelch Like Family Member 5 | 2.09 |
| 51 | * | 253559 | *CADM2* | Cell Adhesion Molecule 2 | 2.07 |
| 52 | * | 4853 | *NOTCH2* | Notch2 | 2.06 |
| 53 | | 6383 | *SDC2* | Syndecan 2 | 2.02 |

* Genes with commercially available antibodies for MACS purposes.
The vmDA cell surface markers identified in this study are shown in red.

Supplementary Table 2. PCR primer sequences for genotyping.

| Primer | | Sequence (5' to 3') | Length | Tm (℃) | Product Size (bp) |
|---|---|---|---|---|---|
| LMX1A-eGFP | LA-F | GCC TAC TTT CCC ATA TCA CAC | 21-mer | 54 | 1,583 |
| | LA-R | CTC CTT GAA GTC GAT GCC C | 19-mer | 56 | |
| | RA-F | GCT TCA AAA GCG CAC GT | 17-mer | 55 | 2,077 |
| | RA-R | CCA ACC CTG CTC TAG AAC AC | 20-mer | 56 | |
| | KI-F | GTA AAT TAG TTG AAG GTA GGC ACC | 24-mer | 54 | 3,599 (KI*) |
| | KI-R | TTG CTG TAC AGT TGG TGT C | 19-mer | 53 | 1,158 (WT**) |

*, knock-in; **, wild type

Supplementary Table 3. Primary antibodies.

| Protein | Species | Company | Cat. no. | Dilution |
|---|---|---|---|---|
| 5-HT | Rabbit | ImmunoStar | 20080 | 1:10,000 |
| ALCAM | Mouse | R&D Systems | MAB561 | 2.5 µg/$10^6$ cells |
| BRACHYURY | Goat | R&D Systems | AF2085 | 1:100 |
| CALB | Rabbit | Millipore | AB1778 | 1:1,000 |
| CD47 | Mouse | Santa Cruz | sc-12730 | 1.0 µg/$10^6$ cells |
| CORIN | Mouse | R&D Systems | MAB2209 | 2.5 µg/$10^6$ cells |
| eGFP | Goat | Rockland | 600-101-215 | 1:1,000 |
| eGFP | Mouse | Rockland | 600-301-215 | 1:1,000 |
| EN1 | Mouse | Dev. Stud. Hybridoma Bank | 4G11 | 1:50 |
| FOXA2 (HNF3β) | Rabbit | Abcam | AB108422 | 1:300 |
| FOXA2 (HNF3β) | Goat | Santa Cruz | sc-6554 | 1:100 |
| GFAP | Rabbit | Millipore | ab5804 | 1:500 |
| GIRK2 | Rabbit | Almone Labs | APC-006 | 1:500 |
| GLU | Rabbit | Sigma-Aldrich | G6642 | 1:2,000 |
| HNA | Mouse | Millipore | MAB1281 | 1:100 |
| Ki67 | Rabbit | Vision Biosystem | NCL-K67P | 1:1,000 |
| LMX1A | Goat | Santa Cruz | sc-54273 | 1:100 |
| LMX1A/B | Rabbit | Millipore | AB10533 | 1:5,000 |
| NANOG (human) | Goat | R&D Systems | AF1997 | 1:50 |
| NCAM (human) | Mouse | Santa Cruz | sc-106 | 1:100 |
| NESTIN (human) | Rabbit | Millipore | ABD69 | 1:1,000 |
| NURR1 | Rabbit | Santa Cruz | sc-990 | 1:1,000 |
| OCT4 | Rabbit | Santa Cruz | sc-9081 | 1:200 |
| PAX6 | Mouse | NOVUS | NBP2-34705AF488 | 1:100 |
| PCNA | Rabbit | Abcam | ab18197 | 1:700 |
| PH3 | Rabbit | Millipore | 06-570 | 1:500 |

(*Continued*)

| Protein | Species | Company | Cat. no. | Dilution |
|---|---|---|---|---|
| PITX3 | Rabbit | NOVUS | NBP1-92274 | 1:500 |
| SMAα | Mouse | Sigma-Aldrich | A5228 | 1:100 |
| SOX1 | Goat | R&D Systems | AF3369 | 1:100 |
| SOX2 | Rabbit | Millipore | AB5603 | 1:200 |
| SSEA4 | Mouse | Millipore | MAB4304 | 1:200 |
| Synapsin 1 | Rabbit | Synaptic System | 106 103 | 1:500 |
| TH | Rabbit | Pel-freez | P40101-0 | 1:1,000 |
| TH | Sheep | Pel-freez | P60101-150 | 1:400 |
| TPBG | Mouse | R&D Systems | MAB49751 | 2.5 μg/$10^6$ cells |
| TRA-1-60 | Mouse | Millipore | MAB4360 | 1:100 |
| TRA-1-81 | Mouse | Millipore | MAB4381 | 1:100 |
| TUJ1 | Mouse | Covance (BioLegend) | MMS-435P (801201) | 1:1,000 |

Supplementary Table 4. qRT-PCR primer sequences.

| Symbol | Gene name | | Sequence (5' to 3') | Tm (℃) |
|---|---|---|---|---|
| *eGFP* | Enhanced green fluorescent protein | F | CAT CAA GGT GAA CTT CAA GAT CCG CCA CAA C | 67.9 |
| | | R | CTT GTA CAG CTC GTC CAT GCC GAG AGT GAT C | 69.7 |
| *EN1* | Engrailed 1 | F | CGT GGC TTA CTC CCC ATT TA | 57.3 |
| | | R | TCT CGC TGT CTC TCC CTC TC | 60.1 |
| *FOXA1* | Forkhead box A1 | F | GGG CAG GGT GGC TCC AGG AT | 66.4 |
| | | R | TGC TGA CCG GGA CGG AGG AG | 65.9 |
| *FOXA2* | Forkhead box A2 (HNF-3β) | F | CCG TTC TCC ATC AAC AAC CT | 57.8 |
| | | R | GGG GTA GTG CAT CAC CTG TT | 59.7 |
| *GAPDH* | Glyceraldehyde-3-Phosphate Dehydrogenase | F | CAA TGA CCC CTT CAT TGA CC | 56.4 |
| | | R | TTG ATT TTG GAG GGA TCT CG | 55.1 |
| *LHX1* | LIM homeobox 1 | F | AGG TGA AAC ACT TTG CTC CG | 58.7 |
| | | R | CTC CAG GGA AGG CAA ACT CT | 59.3 |
| *LMX1A* | LIM homeobox transcription factor 1a | F | CGC ATC GTT TCT TCT CCT CT | 57.7 |
| | | R | CAG ACA GAC TTG GGG CTC AC | 60.3 |
| *LMX1B* | LIM homeobox transcription factor 1b | F | CTT AAC CAG CCT CAG CGA CT | 59.8 |
| | | R | TCA GGA GGC GAA GTA GGA AC | 58.8 |
| *NANOG* | Nanog homeobox | F | TGA ACC TCA GCT ACA AAC AG | 55.3 |
| | | R | TGG TGG TAG GAA GAG TAA AG | 53.7 |
| *NKX2.2* | NK2 homeobox 2 | F | CCT TCT ACG ACA GCA GCG ACA A | 62.8 |
| | | R | ACT TGG AGC TTG AGT CCT GAG G | 61.7 |
| *NKX6.1* | NK6 homeobox 1 | F | CGA GTC CTG CTT CTT CTT GG | 58.2 |
| | | R | GGG GAT GAC AGA GAG TCA GG | 58.9 |
| *OCT4* | POU class 5 homeobox 1 | F | CCT CAC TTC ACT GCA CTG TA | 57.2 |
| | | R | CAG GTT TTC TTT CCC TAG CT | 54.9 |
| *REX1* | ZFP42 zinc finger protein | F | TCA CAG TCC AGC AGG TGT TTG | 60.8 |
| | | R | TCT TGT CTT TGC CCG TTT CT | 57.7 |
| *SIM1* | Single-minded homolog 1 | F | AAA GGG GGC CAA ATC CCG GC | 65.6 |
| | | R | TCC GCC CCA CTG GCT GTC AT | 66.2 |
| *SOX2* | SRY-box2 | F | TTC ACA TGT CCC AGC ACT ACC AGA | 63.6 |
| | | R | TCA CAT GTG TGA GAG GGG CAG TGT GC | 68.8 |
| *SOX6* | SRY-box6 | F | GCC TAA GTG ACC GTT TTG GCA G | 62.3 |
| | | R | GGC ATC TTT GCT CCA GGT GAC A | 63.1 |
| *TET1* | TET methylcytosine dioxygenase 1 | F | CTG CAG CTG TCT TGA TCG AGT TAT | 61.0 |
| | | R | CCT TCT TTA CCG GTG TAC ACT ACT | 59.8 |
| *TPBG* | Trophoblast glycoprotein | F | CAA GGT CCT TCA CAA TGG CAC C | 62.0 |
| | | R | CCT CTG TTT CCT TGA GCC AGG T | 62.2 |
