# Supplementary material for: Trophoblast glycoprotein is a marker for efficient sorting of ventral mesencephalic dopaminergic precursors derived from human pluripotent stem cells
Source: NPJ Parkinsons Dis. 2021 Jul 19;7:61. doi: 10.1038/s41531-021-00204-8 (PMC8289854; doi:10.1038/s41531-021-00204-8)
Supplement: Supplementary file 2 — Reporting Summary [file 41531_2021_204_MOESM2_ESM.pdf]

nature portfolio

Corresponding author(s): Dong-Wook Kim, Dong-Youn Hwang

Last updated by author(s): Jun 18, 2021

Reporting Summary

Nature Portfolio wishes to improve the reproducibility of the work that we publish. This form provides structure for consistency and transparency in reporting. For further information on Nature Portfolio policies, see our Editorial Policies and the Editorial Policy Checklist.

Statistics

For all statistical analyses, confirm that the following items are present in the figure legend, table legend, main text, or Methods section.

n/a	Confirmed	
☐	☒	The exact sample size (n) for each experimental group/condition, given as a discrete number and unit of measurement
☐	☒	A statement on whether measurements were taken from distinct samples or whether the same sample was measured repeatedly
☐	☒	The statistical test(s) used AND whether they are one- or two-sided<br>*Only common tests should be described solely by name; describe more complex techniques in the Methods section.*
☐	☒	A description of all covariates tested
☐	☒	A description of any assumptions or corrections, such as tests of normality and adjustment for multiple comparisons
☐	☒	A full description of the statistical parameters including central tendency (e.g. means) or other basic estimates (e.g. regression coefficient) AND variation (e.g. standard deviation) or associated estimates of uncertainty (e.g. confidence intervals)
☐	☒	For null hypothesis testing, the test statistic (e.g. F, t, r) with confidence intervals, effect sizes, degrees of freedom and P value noted<br>*Give P values as exact values whenever suitable.*
☒	☐	For Bayesian analysis, information on the choice of priors and Markov chain Monte Carlo settings
☒	☐	For hierarchical and complex designs, identification of the appropriate level for tests and full reporting of outcomes
☒	☐	Estimates of effect sizes (e.g. Cohen's d, Pearson's r), indicating how they were calculated

Our web collection on statistics for biologists contains articles on many of the points above.

Software and code

Policy information about availability of computer code

Data collection	FACSDiva softare (BD), Olympus FSX100 system, LSM710 confocal microscope, Bio-Rad CFX Manager v3.1, Affymetrix GCS3000 Scanner
Data analysis	Fiji-ImageJ 1.53c, ZEN 3.1, FlowJo software v10, Affymetrix® Expression Console™ Software, ImageScope software v12.3.0.506, GraphPad Prism v7.00

For manuscripts utilizing custom algorithms or software that are central to the research but not yet described in published literature, software must be made available to editors and reviewers. We strongly encourage code deposition in a community repository (e.g. GitHub). See the Nature Portfolio guidelines for submitting code & software for further information.

Data

Policy information about availability of data

All manuscripts must include a data availability statement. This statement should provide the following information, where applicable:

- Accession codes, unique identifiers, or web links for publicly available datasets
- A description of any restrictions on data availability
- For clinical datasets or third party data, please ensure that the statement adheres to our policy

All data generated or analyzed during this study are included in this published article (and its supplementary information files). The microarray data that support the findings of this study have been deposited in GEO with the accession codes GSE171769.

Field-specific reporting

Please select the one below that is the best fit for your research. If you are not sure, read the appropriate sections before making your selection.

☒ Life sciences ☐ Behavioural & social sciences ☐ Ecological, evolutionary & environmental sciences

For a reference copy of the document with all sections, see nature.com/documents/nr-reporting-summary-flat.pdf

Life sciences study design

All studies must disclose on these points even when the disclosure is negative.

Sample size	No statistical analysis was employed to determine the sample size. The sample size was chosen base on previous publications using the same techniques.
Data exclusions	No data were excluded.
Replication	All statistical analysis were based on at least three replicates. More details of biological repeats are described in each figure legend.
Randomization	Images from Immunocytochemistry were captured randomly, however all positive signals were analyzed equally under same conditions.
Blinding	Immunohistochemical and behavioral analyses in the animal study were conducted and analyzed by experiments blinded to the groups.

Reporting for specific materials, systems and methods

We require information from authors about some types of materials, experimental systems and methods used in many studies. Here, indicate whether each material, system or method listed is relevant to your study. If you are not sure if a list item applies to your research, read the appropriate section before selecting a response.

Materials & experimental systems

n/a	Involved in the study	
☐	☒	Antibodies
☐	☒	Eukaryotic cell lines
☒	☐	Palaeontology and archaeology
☐	☒	Animals and other organisms
☒	☐	Human research participants
☒	☐	Clinical data
☒	☐	Dual use research of concern

Methods

n/a	Involved in the study	
☒	☐	ChIP-seq
☐	☒	Flow cytometry
☒	☐	MRI-based neuroimaging

Antibodies

Antibodies used	We provide the all the essential information for antibodies in Supplementary Table 3.
Validation	The antibodies used were validated by the previous publications or routine experimental protocols, listed in Supplementary Table 3.

Eukaryotic cell lines

Policy information about cell lines

Cell line source(s)	H9 (WA09) hESCs were purchased from WiCell Inc. (USA). In this study, we generated reporter hESC lines in which an eGFP reporter gene is inserted into the LMX1A locus via TALEN-mediated genome editing.
Authentication	The cell lines used were checked for morphology by microscopy and immunostaining with specific markers.
Mycoplasma contamination	We periodically checked potential contamination with mycoplasma. All cell lines tested negative for mycoplasma contamination using e-Myco Mycoplasma PCR Detection Kit (ver.2.0).
Commonly misidentified lines (See ICLAC register)	No. The cell lines used are not listed in the database.

Animals and other organisms

Policy information about studies involving animals; ARRIVE guidelines recommended for reporting animal research

Laboratory animals	Female Sparague-Dqwley (SD) rats of 6 to 29 weeks of age were used for experiments.
Wild animals	This study does not involve wild animals.
Field-collected samples	This study does not involve samples collected from the field.
Ethics oversight	All experimental procedures for this study were approved by the Institutional Animal Care and Use Committee of the Yonsei University Health System (2016-0088; 2019-0087).

Note that full information on the approval of the study protocol must also be provided in the manuscript.

Flow Cytometry

Plots

Confirm that:

☒ The axis labels state the marker and fluorochrome used (e.g. CD4-FITC).

☒ The axis scales are clearly visible. Include numbers along axes only for bottom left plot of group (a 'group' is an analysis of identical markers).

☒ All plots are contour plots with outliers or pseudocolor plots.

☒ A numerical value for number of cells or percentage (with statistics) is provided.

Methodology

Sample preparation	We provide the all the essential information for sample preparation and immunostaining for flow cytometry in the 'Methods' part.
Instrument	FACS was performed using a BD FACSAria-III cell sorter and FACSDiva software (BD Biosciences). Flow cytometry was performed using LSRII (BD Biosciences), and analysis was performed using FlowJo software (FlowJo).
Software	FACSDiva software (BD Biosciences), FlowJo software (FlowJo)
Cell population abundance	Purity was determined by running a purity check of the sorted populations after the sort was completed.
Gating strategy	All samples were initially gated using forward scatter and side scatter to identify events corresponding to cells, and then using forward scatter height vs. area to enrich for single cells. The follow gaiting steps are presented in Methods and Supplementary Fig. 14.

☒ Tick this box to confirm that a figure exemplifying the gating strategy is provided in the Supplementary Information.
